# Genomic Diversity and Evolution of the Fish Pathogen *Flavobacterium psychrophilum*

**DOI:** 10.3389/fmicb.2018.00138

**Published:** 2018-02-07

**Authors:** Eric Duchaud, Tatiana Rochat, Christophe Habib, Paul Barbier, Valentin Loux, Cyprien Guérin, Inger Dalsgaard, Lone Madsen, Hanne Nilsen, Krister Sundell, Tom Wiklund, Nicole Strepparava, Thomas Wahli, Greta Caburlotto, Amedeo Manfrin, Gregory D. Wiens, Erina Fujiwara-Nagata, Ruben Avendaño-Herrera, Jean-François Bernardet, Pierre Nicolas

**Affiliations:** ^1^Unité de Virologie et Immunologie Moléculaires (VIM), Institut National de la Recherche Agronomique, Université Paris-Saclay, Jouy-en-Josas, France; ^2^Unité Mathématiques et Informatique Appliquées du Génome à l'Environnement (MaIAGE), Institut National de la Recherche Agronomique, Université Paris-Saclay, Jouy-en-Josas, France; ^3^Section for Bacteriology and Pathology, National Veterinary Institute, Technical University of Denmark, Kgs. Lyngby, Denmark; ^4^Department of Aquatic Animal health, Norwegian Veterinary Institute, Bergen, Norway; ^5^Laboratory of Aquatic Pathobiology, Environmental and Marine Biology, Faculty of Science and Engineering, Åbo Akademi University, Turku, Finland; ^6^Laboratory of Applied Microbiology, Department for Environment Constructions and Design, University of Applied Sciences and Arts of Southern Switzerland (SUPSI), Bellinzona, Switzerland; ^7^Centre for Fish and Wildlife Health (FIWI), University of Bern, Bern, Switzerland; ^8^Department of Fish Pathology, Istituto Zooprofilattico Sperimentale delle Venezie, Legnaro, Italy; ^9^National Center for Cool and Cold Water Aquaculture, Agricultural Research Service, United States Department of Agriculture, Kearneysville, WV, United States; ^10^Department of Fisheries, Kindai University, Nara, Japan; ^11^Departamento Facultad de Ciencias Biológicas, Universidad Andres Bello, Universidad Andres BelloViña del Mar, Interdisciplinary Center for Aquaculture Research, Concepción, Chile

**Keywords:** *Flavobacterium psychrophilum*, aquaculture, fish-pathogenic bacteria, comparative genomics, clonal-complex, homologous recombination

## Abstract

*Flavobacterium psychrophilum*, the etiological agent of rainbow trout fry syndrome and bacterial cold-water disease in salmonid fish, is currently one of the main bacterial pathogens hampering the productivity of salmonid farming worldwide. In this study, the genomic diversity of the *F. psychrophilum* species is analyzed using a set of 41 genomes, including 30 newly sequenced isolates. These were selected on the basis of available MLST data with the two-fold objective of maximizing the coverage of the species diversity and of allowing a focus on the main clonal complex (CC-ST10) infecting farmed rainbow trout (*Oncorhynchus mykiss*) worldwide. The results reveal a bacterial species harboring a limited genomic diversity both in terms of nucleotide diversity, with ~0.3% nucleotide divergence inside CDSs in pairwise genome comparisons, and in terms of gene repertoire, with the core genome accounting for ~80% of the genes in each genome. The pan-genome seems nevertheless “open” according to the scaling exponent of a power-law fitted on the rate of new gene discovery when genomes are added one-by-one. Recombination is a key component of the evolutionary process of the species as seen in the high level of apparent homoplasy in the core genome. Using a Hidden Markov Model to delineate recombination tracts in pairs of closely related genomes, the average recombination tract length was estimated to ~4.0 Kbp and the typical ratio of the contributions of recombination and mutations to nucleotide-level differentiation (r/m) was estimated to ~13. Within CC-ST10, evolutionary distances computed on non-recombined regions and comparisons between 22 isolates sampled up to 27 years apart suggest a most recent common ancestor in the second half of the nineteenth century in North America with subsequent diversification and transmission of this clonal complex coinciding with the worldwide expansion of rainbow trout farming. With the goal to promote the development of tools for the genetic manipulation of *F. psychrophilum*, a particular attention was also paid to plasmids. Their extraction and sequencing to completion revealed plasmid diversity that remained hidden to classical plasmid profiling due to size similarities.

## Introduction

Aquaculture is the fastest growing food-producing sector and now plays a critical role in global food supply. Salmonids (salmons and trouts) represent an essential group of fish in this industry (The state of world fisheries and aquaculture 2016, FAO). However, the rapid development of intensive aquaculture has been associated with a dramatic increase in infectious diseases outbreaks. In this context, the success and sustainability of salmonid aquaculture largely depends on the control of fish pathogens. Among those, *Flavobacterium psychrophilum*, a Gram-negative bacterium of the family *Flavobacteriaceae* in the phylum *Bacteroidetes* (Bernardet and Bowman, 2006), is widely recognized as one of the main sources of disease and economic loss for the salmonid industry worldwide (Barnes and Brown, 2011; Starliper, 2011).

All salmonid fish found in freshwater appear susceptible to *F. psychrophilum* infections, especially coho salmon (*Oncorhynchus kisutch*), rainbow trout (*Oncorhynchus mykiss*), Atlantic salmon (*Salmo salar*), and ayu (*Plecoglossus altivelis*), a fish related to salmonids. Annually, the two main clinical forms of *F. psychrophilum* infection, which are rainbow trout fry syndrome (RTFS) and bacterial cold-water disease (BCWD) (Nematollahi et al., 2003; Cipriano and Holt, 2005), result in millions of dollars in deficits for anglers and hatcheries (Antaya, 2008). The control of these diseases is complicated by the multiple modes of propagation of *F. psychrophilum* which can survive in freshwater for long periods (Madetoja et al., 2003) and is not only horizontally transmitted but also vertically or pseudo-vertically transmitted (Brown et al., 1997; Madsen and Dalsgaard, 2008; Kumagai and Nawata, 2010a). Moreover, egg disinfection using the standard povidone-iodine treatment is poorly efficient, making this route of pathogen propagation particularly difficult to control (Taylor, 2004; Kumagai and Nawata, 2010b; Grasteau et al., 2015). Despite extensive research on vaccine development (Plant et al., 2009; Gómez et al., 2014) and the recent marketing authorization of a vaccine in some countries, treatment still essentially relies on antibiotic administration (Schmidt et al., 2000).

Historically, the first cases of infections with *F. psychrophilum* were reported in North America in the 1930s. The pathogen was repeatedly reported since then but only in North America until the mid-1980s when the bacterium was identified in France, Germany, Denmark, and Japan (Borg, 1948; Bernardet and Kerouault, 1989; Lorenzen et al., 1991; Wakabayashi et al., 1991). It has later been found in Chile (Bustos et al., 1995) and all regions of the world involved in salmonid aquaculture (Starliper, 2011). *F. psychrophilum* has occasionally been retrieved from feral salmonids with or without clinical signs (Fujiwara-Nagata et al., 2013; Van Vliet et al., 2016), non-salmonid fish, samples of water, sediments, and biofilms from rivers receiving outlet water from infected fish farms (Madetoja and Wiklund, 2002; Starliper, 2011).

Genetic diversity among *F. psychrophilum* isolates started to be studied in the early 1990s using an array of methods such as random amplification of polymorphic DNA, PCR-restriction fragment length polymorphism, ribotyping, pulsed-field gel electrophoresis, serotyping, and plasmid profiling. Later, the development of a MLST scheme (Nicolas et al., 2008) and its further use at a larger scale in different geographical contexts has provided insights into the molecular diversity of this species and allowed resolution between several epidemiological scenario hypotheses (Siekoula-Nguedia et al., 2012; Fujiwara-Nagata et al., 2013; Strepparava et al., 2013; Avendaño-Herrera et al., 2014; Nilsen et al., 2014; Van Vliet et al., 2016). Strikingly, an epidemic population structure containing a limited number of clonal complexes with marked association with host fish species was observed. Fish trade is suspected to have allowed the transcontinental dissemination of some epidemic clones. In particular, bacterial isolates belonging to CC-ST10 group (renamed from CC-ST2 by Nilsen et al., 2014) were retrieved almost exclusively from farmed rainbow trout (Van Vliet et al., 2016).

Since the publication of the first *F. psychrophilum* complete genome (Duchaud et al., 2007) sequences from only a limited number of additional isolates have been published (Wiens et al., 2014; Wu et al., 2015; Castillo et al., 2016; Neiger et al., 2016; Shimizu et al., 2016; Rochat et al., 2017a) and a global comparative genomics analysis of the species is missing. Here, we report the draft genome sequencing of 30 additional isolates selected on the basis of their origins and MLST profiles. These data allowed a comparison of 41 *F. psychrophilum* isolates representing a diversity of host fish species, geographical areas and years of isolation. They are used here to draw a global picture of the genomic diversity of the species and to characterize the evolutionary processes that shape this diversity at different time-scales. In particular, we compare *F. psychrophilum* genomes in terms of gene content, analyze the allele frequency spectrum of the SNPs and the impact of recombination, and investigate the evolution of CC-ST10.

## Materials and methods

### DNA extraction

Cultures of each *F. psychrophilum* isolate were grown at 18°C and 200 rpm in tryptone yeast extract salts (TYES) broth (0.4% tryptone, 0.04% yeast extract, 0.05% MgSO_4_ 7H_2_O, 0.02% CaCl_2_ 2H_2_O, 0.05% D-glucose, pH 7.2).

Genomic DNA used for high-throughput DNA sequencing was extracted using a Wizard genomic DNA purification kit (Promega). Plasmid DNA was prepared using alkaline lysis as follows: 2 mL of OD600 ~2 bacterial culture was centrifuged, the pellet resuspended in 0.2 mL of buffer (Tris 50 mM pH 8, 20% glucose, EDTA 10 mM, lysozyme 2 mg mL^−1^, RNase A 1 mg mL^−1^) and incubated for 20 min at 37°C. After addition of 0.4 mL lysis buffer (NaOH 0.2 N, 1% SDS) and incubation at 56°C for 20 min, 0.3 mL of potassium acetate 3M—glacial acetic acid 2M solution was added and the mixture incubated on ice for 20 min before centrifugation during 10 min at 13,000 rpm 4°C. The supernatant was then phenol-chloroform extracted and the aqueous phase transferred to a new tube.

Plasmid DNA was recovered by precipitation using isopropanol and sodium acetate pH 6. After centrifugation, the pellet was dissolved in TE. These DNA extracts were used for electrophoresis on TAE 1% gels or as DNA templates for PCR.

### Genome sequences and annotation

The 30 *F. psychrophilum* genomes sequenced in this study were obtained using Illumina technology (GA2 and HiSeq, single reads and paired-end) and the reads were processed using workflows relying on available tools. For all sequences in Supplementary Table 1 excepted FRGDSA 1882/11, the building blocks of the workflow were the following: FastQC (v0.10.0) for quality assessment; an home-made Perl script for trimming each read by removing 5′ and 3′ terminal positions of Sanger quality <10 and until reaching an average quality ≥20 without N; Velvet (v1.2.07) (Zerbino and Birney, 2008) and VelvetOptimiser (v2.2.0) for assembly based on de Bruijn graphs with an optimized k-mer length (selected k-mer length varied from 31 for KU 061128-1 to 71 for IT02); the average insert size estimated by mapping using BWA (v0.7.5a-r405) (Li and Durbin, 2010) on the JIP 02/86 complete genome was provided to Velvet for paired-end data sets (option -ins_length, here between 230 and 340 bp). For FRGDSA 1882/11, which was the last genome added to our collection, we used a slightly updated workflow: read trimming relied on Sickle (v1.33) and assembly on Velvet (v1.2.08) without prior estimation of the insert size; the selected k-mer length was 97.

In link with the year of sequencing, read-length, depth of sequencing and consequently the number of contigs varied between genomes (Supplementary Table 1). Annotation of protein coding genes was performed using the MicroScope platform (Vallenet et al., 2017). Manual inspection and annotation was performed in relevant, selected regions. Annotated genome sequences were deposited in EMBL, accession numbers are reported in Table 1.

**Table 1:** Genome sequences included in the comparative analysis.

**Isolate**	**Contigs^a^**	**Kbp^b^**	**Country^c^**	**Fish host^d^**	**Tissue**	**Year**	**Publication^e^**	**Accession Number**
JIP 02/86	1	2,860	France	*O. mykiss*	Kidney	1986	Duchaud et al., 2007	AM398681.2
CSF259-93	1	2,901	ID, USA	*O. mykiss*	Spleen	1993	Wiens et al., 2014	CP007627
NCIMB 1947^T^	1	2,716	WA, USA	*O. kisutch*	Kidney	n.a.^i^	Wu et al., 2015	CP007207
DIFR 950106-1/1	1	2,736	Denmark	*O. mykiss*^g^	Spleen	1995	Castillo et al., 2016	SAMN02903962
FPG101	1	2,835	Canada	*O. mykiss*	n.a.	2008	Castillo et al., 2016	SAMN03290989
MH1	1	2,848	Chile	*Salmo salar*	Skin	2008	Castillo et al., 2016	SAMN02941921
PG2	1	2,851	Chile	*O. mykiss*	Skin	2009	Castillo et al., 2016	SAMN02941922
5	1	2,848	Chile	Fish farm freshwater		2013	Castillo et al., 2016	SAMN02941920
3	1	2,805	Chile	Fish farm freshwater		2013	Castillo et al., 2016	SAMN02941918
VQ50	1	2,807	Chile	*O. mykiss*	Skin	2006^j^	Castillo et al., 2016	SAMN02941923
OSU THCO2-90^f^	1	2,784	OR, USA	*O. kisutch*	Kidney	1990	Rochat et al., 2017a	LT670843
KU 051128-10	179	2,572	Japan	River water		2005	This study (EFN)	GCA_900186555
KU 060626-4	182	2,527	Japan	*P. altivelis*	Kidney	2006	This study (EFN)	GCA_900186475
KU 060626-59	204	2,518	Japan	*P. altivelis*	Skin lesion	2006	This study (EFN)	GCA_900186495
KU 061128-1	202	2,515	Japan	River water		2006	This study (EFN)	GCA_900186605
IT02	43	2,648	Italy	*O. mykiss*	Spleen	2011	This study (GC)	GCA_900186545
IT09	46	2,716	Italy	*O. mykiss*	Spleen	2012	This study (GC)	GCA_900186525
NO004	58	2,774	Norway	*Salmo trutta*	Fin	1998	This study (HN)	GCA_900186755
NO014	66	2,682	Norway	*O. mykiss*	Spleen	2008	This study (HN)	GCA_900186785
NO042	73	2,583	Norway	*Salmo salar*	Spleen	2008	This study (HN)	GCA_900186825
NO083	42	2,690	Norway	*O. mykiss*	Kidney	2011	This study (HN)	GCA_900186805
NO098	52	2,617	Norway	*Salmo salar*	Ovarian fluid	2011	This study (HN)	GCA_900186815
DK002	40	2,686	Denmark	*O. mykiss*	kidney	1990	This study (ID)	GCA_900186425
DK150	66	2,775	Denmark	*O. mykiss*	Kidney	1995	This study (ID)	GCA_900186365
DK095	66	2,816	Denmark	*G. aculeatus*	Skin	2000	This study (ID)	GCA_900186385
DK001	48	2,704	Denmark	*O. mykiss*	Spleen	2009	This study (ID)	GCA_900186405
FPC 840	274	2,516	Japan	*P. altivelis*	Kidney	1987	This study (J-FB)	GCA_900186575
FPC 831	226	2,600	Japan	*O. kisutch*	Peduncle lesion	1990	This study (J-FB)	GCA_900186485
LVDJ XP189	258	2,685	France	*Tinca tinca*	Kidney	1992	This study (J-FB)	GCA_900186645
JIP 08/99	261	2,549	France	*O. mykiss*	Kidney	1999	This study (J-FB)	GCA_900186595
JIP 16/00	368	2,503	France	*O. mykiss*	n.a.	2000	This study (J-FB)	GCA_900186515
FRGDSA 1882/11	74	2,687	France	*O. mykiss*	n.a.	2011	This study (J-FB)	GCA_900186415
CH8	58	2,699	Switzerland	*O. mykiss*	Spleen	2009	This study (NS)	GCA_900186345
CH1895	65	2,748	Switzerland	*Salmo trutta*^h^	Skin	2011	This study (NS)	GCA_900186435
LM-01-Fp	46	2,753	Chile	*O. mykiss*	Kidney	2006	This study (RA-H)	GCA_900186685
LM-02-Fp	43	2,685	Chile	*O. mykiss*	Kidney	2006	This study (RA-H)	GCA_900186665
FI055	78	2,732	Finland	*O. mykiss*	Inner organs	1996	This study (TW)	GCA_900186395
FI056	41	2,694	Finland	*O. mykiss*	inner organs	1996	This study (TW)	GCA_900186375
FI146	99	2,838	Finland	Pond water		2000	This study (TW)	GCA_900186455
FI070	52	2,715	Finland	*P. fluviatilis*	Mouth	2006	This study (TW)	GCA_900186445
FI166	62	2,732	Scotland	*Salmo salar*	n.a.	2007	This study (TW)	GCA_900186565

a *Number of contigs (≥ 1 Kbp, plasmids excluded)*.

b *Cumulated contig length*.

c *The names of USA states are abbreviated*.

d *Abbreviated species names for Gasterosteus aculeatus, Oncorhynchus mykiss, Oncorhynchus kisutch, Perca fluviatilis, Plecoglossus altivelis*.

e *Initials of contact name for newly sequenced isolates; Erina Fujiwara-Nagata, Greta Caburlotto, Hanne Nilsen, Inger Daslgaard, Jean-François Bernardet, Nicole Strepparava, Ruben Avendaño-Herrera, Tom Wiklund. n.a., no data available*.

f *The spelling OSU THC02-90 has been used in several publications (e.g., Nicolas et al., 2008) but the original name of this strain is OSU THCO2-90 (Bertolini et al., 1994)*.

g *Information on host fish for DIFR 950106-1/1 can be found in Madsen and Dalsgaard (1999)*.

h *S. trutta lacustris*.

i *The exact date of isolation of the type strain NCIMB 1947^T^ is unknown; it was most likely isolated by E.J. Ordal sometime between 1945 and 1960*.

j *VQ50 was isolated by R. Avendaño-Herrera in 2006 in Veterquímica (VQ)*.

As already performed for isolate OSU THCO2-90 (Rochat et al., 2017a), optical maps, using the Argus system (OpGene, Maryland, USA) and the *Spe*I restriction enzyme, were performed for strains JIP 02/86 (also known as ATCC 49511) and NCIMB 1947^T^ (aka ATCC 49418^T^).

### Plasmid sequences

The repertoire of plasmid sequences in *F. psychrophilum* was established by screening of assembled contigs for signature genes (i.e., genes encoding replicase and toxin/antitoxin systems). When needed to close gaps or to confirm plasmid sequences, PCR followed by Sanger sequencing was carried out and the resulting reads were assembled using phred-phrap-consed (Ewing et al., 1998). For assembly validation, plasmid sequence lengths were compared with relative migration measured by gel electrophoresis.

### Gene families

Gene families were built using a Single-Linkage criterion (Lerat et al., 2003) based on the results of pairwise proteome comparisons using blastp (v2.2.6) with an E-value cut-off set to 1e-5. Blast hits established a link between two genes when the percentage of amino-acid sequence identity in the alignment was greater than 85% and when the length of the alignment accounted for at least 70% of the longest sequence. To evaluate the number of distinct gene families in the pan-genome we applied a relaxed cut-off on alignment length, which consisted in requiring it to account for 70% of the shortest sequence. By doing this, we tried to minimize the chances of counting truncated sequences as new families since these typically arise from pseudogenization events, sequencing errors and gaps between contigs.

### Whole-genome multi-locus sequence typing and SNP calling

Within each gene family, protein sequences were subjected to multiple sequence alignment with Muscle (v3.8.31, default parameters) (Edgar, 2004). In order to avoid artifactual polymorphism caused by inconsistent start codon prediction or gaps between contigs, the resulting protein sequence alignments were truncated on the N-term and C-term sides to ensure flanking by at least three perfectly conserved amino-acids. The truncated protein sequence alignment was then converted into a DNA sequence alignment before examining nucleotide polymorphism.

Unique identifiers were attributed to the distinct allele types (ATs) defined on the basis of the multiple DNA sequence alignments. This is in keeping with the practice of whole-genome multi-locus typing (Jolley and Maiden, 2010) which extends the idea of classical multi-locus typing thoroughly used for micro-evolution and population structure analyses based on a small number (typically 7) of conserved loci where polymorphism is likely not to be under strong diversifying selection.

### Phylogenetic tree reconstructions

The high amount of recombination makes detailed phylogenetic reconstructions difficult in *F. psychrophilum* but trees remain very useful to represent the relationships between isolates. Tentative phylogenetic trees were obtained using the Neighbor-Joining algorithm implemented in R library “ape” which also served for tree drawing (Paradis, 2012). Computation of the apparent amount of homoplasy at the nucleotide level used trees reconstructed using the parsimony algorithm implemented in the program “dnapars” of the Phylip package (Felsenstein, 2005). To apply the parsimony method to AT-profiles we relied on parsimony functions (“optim.parsimony” and “acctran”) available in the R library “phangorn” (Schliep, 2011).

### Analysis of recombination rates with hidden markov models

To delineate recombination tracts, we examined pairs of genomes belonging to groups of closely related isolates (in practice AT-divergence within the selected groups was not higher than 30%). Binary nucleotide polymorphisms located in single copy genes were positioned on a synthetic chromosome backbone based on the gene coordinates of isolate CSF259-93. This isolate was chosen because it belongs to ST10, one of the predicted founding genotype of CC-ST10 (Nilsen et al., 2014). Polymorphism was then recoded in a three-letter alphabet (P0, P1, P2): P0 for non-polymorphic positions; P1 for polymorphic sites with one allele specific to the group of closely related isolates; and P2 for polymorphic sites with both alleles also found outside of this group of closely related isolates. Of note, mutation events generate a vast majority of positions in category P1 since the limited nucleotide diversity in the species makes the expected amount of true homoplasy very low. In contrast, most of the nucleotide polymorphism generated by recombination events is expected to belong to category P2.

A Hidden Markov Model (HMM) constituted of two hidden states was used to delineate in an automated and consistent manner two types of regions corresponding to recombination tracts (R) and the non-recombined background (B). Gaps were ignored since they typically do not fulfill the hypothesis of independence between adjacent positions, assumed by the HMM for the emitted character conditionally on the hidden state variable. Data at positions outside of the aligned regions of the conserved CDSs were treated as missing at random. Each hidden state has its own emission probability spectrum for P0, P1, and P2 and the transition probabilities between the two hidden states (two free parameters) allow to describe the frequency and the average length of the recombination tracts. Here, the emission probability for P2 was set to 0 in state B, which led to a total of 5 free parameters that were initially estimated independently for each pair of closely related isolates by likelihood maximization using the EM algorithm implemented in C++ (algorithm described in Nicolas et al., 2002). In the range of evolutionary distance considered here, maximum likelihood estimates of the emission spectrum in state R and the parameter that controls average length in state R exhibited only limited variations (coefficient of variations: 25% for recombination tract length, 33% for the rate of P1, and 16% for the rate of P2) without apparent trend with respect to the degree of relatedness between isolates. Therefore, to further increase the consistency between the estimates obtained for each pair of sequences, the emission spectrum for P1 and P2 in state R were set to 0.00028 and 0.00291 and the expected average length of state R was set to 4,014 bp (values selected as the median of the parameters estimated with 5 free parameters). The two remaining estimated free parameters were the probability of emission of P1 in state B and the transition probability between state B and R. These could be interpreted as the amount of divergence by mutation between the two isolates and the apparent amount of recombination events, respectively. The contribution of recombination and mutation to the divergence between closely related isolates could then be disentangled either by computing their expected values from the estimated parameters or by identifying the SNPs belonging to recombination tracts via reconstruction of the hidden path (forward-backward algorithm).

## Results

### Genome characteristics and gene families

Characteristics of genome sequences and origins of the 41 isolates are listed in Table 1. The newly sequenced genomes were not fully assembled. The numbers of contigs varying from 43 to 368 mainly reflected the depth of sequencing and the properties of the reads (Supplementary Table 1). Genome length, estimated by the cumulated size of the contigs, had a mean of 2,714 Kbp and exhibited little variation between isolates. The corresponding standard deviation was 102 Kbp which over-estimates the biological variability since genome length was negatively correlated to the number of contigs (Pearson correlation −0.74, *p*-value 2.5e-08). Each genome contained an average of 2,442 predicted protein coding sequences (CDSs). In terms of genome structure, the genomes were largely co-linear as illustrated by the conservation of gene chromosomal positions between isolates OSU THCO2-90 and CSF259-93 (Supplementary Figure 1). However, a large chromosomal inversion around the terminus (~1.9 Mbp) flanked by inverted repeats was confirmed between isolates JIP 02/86 and CSF259-93 by optical mapping (Supplementary Figure 1). The 10 Kbp repeated inverted region consists of three genes predicted to encode an adhesin, a Type IX secretion system membrane protein (PorP/SprF family) and an outer membrane protein. This remarkable inversion is reminiscent of the chromosomal flip-flop described in *Staphylococcus aureus* (Cui et al., 2012).

A total of 100,125 CDSs were found in the 41 genomes. The comparative genome analysis started by the delineation of gene families. Considering the low levels of sequence divergence previously reported in *F. psychrophilum* for typical core-genome genes used in MLST (Nicolas et al., 2008), we choose a cut-off of 85% for sequence identity between the aligned portion of two proteins to determine whether two CDSs were members of the same gene family. Functional considerations led us to also use a cut-off of 70% on the minimal coverage of the length of the genes by the alignment. The coverage value on which this cut-off is applied could be computed either with respect of the longest or shortest gene in the aligned pair. When computed with respect to the longest gene, it resulted in 5,859 gene families with a compact distribution of gene lengths (only 2.6% displayed variation in length ≥ 100 aa). When computed with respect to the shortest gene, the number of gene families decreased to 4,805 and the distribution of gene lengths was more variable (10.4% displayed variation in length ≥ 100 aa). This was expected as both pseudogenization events and gaps between contigs can lead to gene fragments that artificially increase the total number of gene families. We used these 4,805 gene families for our analysis of the gene repertoires but preferred the 5,859 gene families with a compact distribution of gene lengths for analyses based on a multiple sequence alignments (analysis of SNPs and allele types). The definition of gene families was globally robust with respect to moderate changes of the cut-off on amino-acid sequence identity (5,781 families when set to 90% vs. 5,986 families when set to 80%).

### Nucleotide diversity and population structure

Our analysis of the nucleotide diversity focused on the 1,549 gene families (out of 5,859) with a single representative in each sequenced genome. Based on multiple sequence alignments obtained for each gene family, we computed the density of SNPs and the density of gaps. Although these densities were generally very low with medians for the density of SNPs and gaps respectively of 0.0095 and 0.0039 per bp, some gene families clearly appeared as outliers presumably due to occasional horizontal gene transfers from closely related species. To capture the diversity resulting of within species evolution and to keep only the highly reliable alignments, we discarded gene families with density of SNPs or gaps > 0.05 per bp (44 and 6 gene families, respectively). This resulted in a set of 1,500 aligned gene families of cumulated length 1,489 Kbp (~54.9% of each genome) encompassing 15,543 SNPs out of which 5,209 are sites of polymorphism at the amino-acid level. The total number of gaps was only 305 and the corresponding sites were not taken into account in the analysis of the nucleotide diversity.

**Figure 1:**
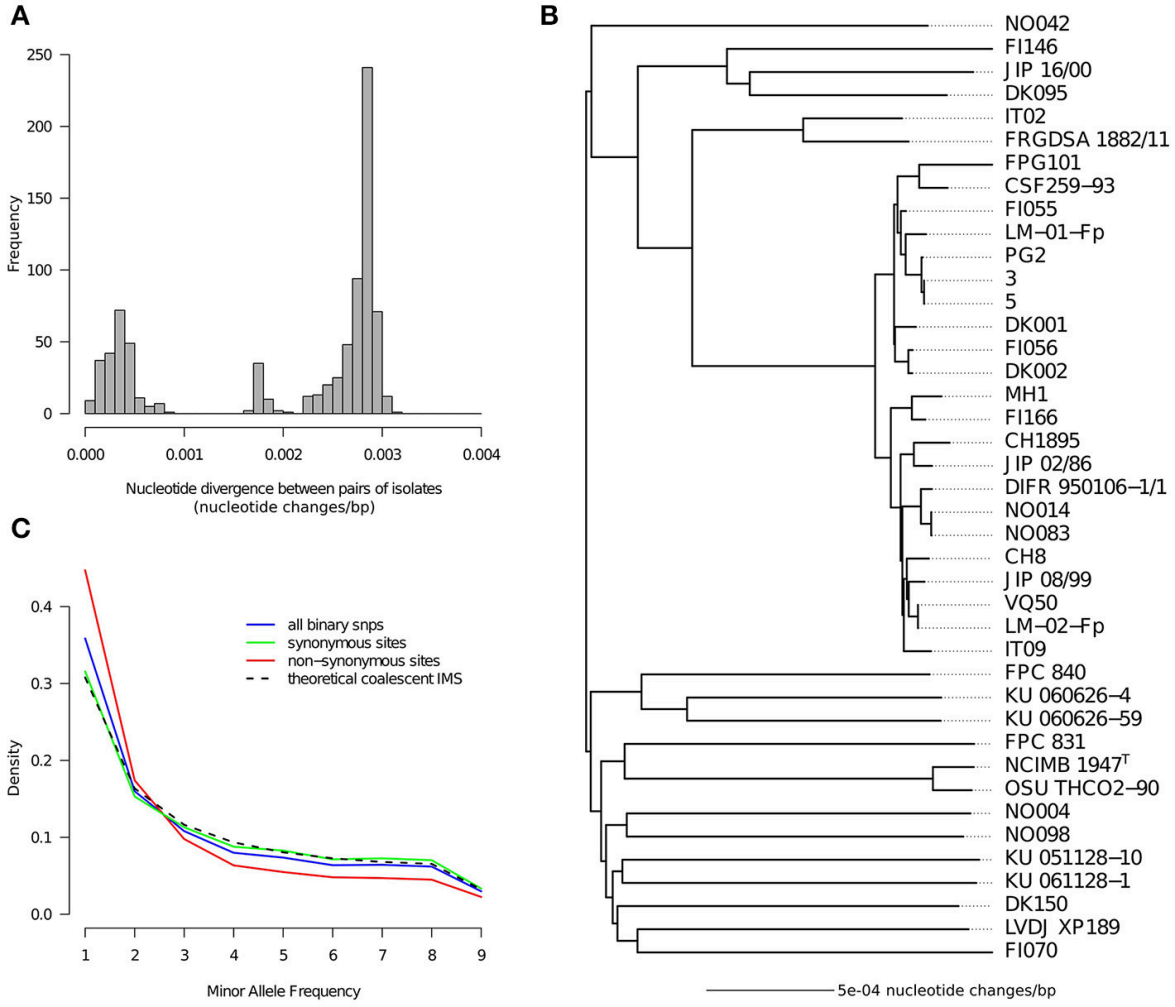
Nucleotide diversity**. (A)** Distribution of pairwise nucleotide distances between genomes. **(B)** Tree representation of pairwise nucleotide distances obtained with the neighbor-joining method. **(C)** Folded nucleotide frequency spectrum for binary SNPs as computed on the 18 isolates obtained when taking only one representative per clonal complex. The spectrum is shown for three categories of sites based on the detection or not of amino-acid polymorphism in the concerned codon: synonymous sites, non-synonymous sites, all sites. The theoretical spectrum under the idealized scenario of the standard coalescent with infinitely many sites is also shown for comparison.

The distribution of pairwise nucleotide divergence between isolates across these 1,489 Kbp of conserved single copy genes is represented in Figure 1A. It exhibits at least two distinct modes as expected from our sampling scheme which intentionally included many isolates belonging to a same clonal complex (CC-ST10). These closely related isolates have pairwise divergences most often below 0.0005 nucleotide changes/bp (and always below 0.001) whereas the pairwise nucleotide divergence for the general population peaks at 0.00285 nucleotide changes/bp.

A tree was built to depict the relationships between the isolates captured in the pairwise divergences (Figure 1B). As anticipated from the relatively homogeneous level of divergence between isolates belonging to different clonal complexes, the tree tends to have a star-like shape where lineages coalesce near the root. Consequently, outside clonal complexes, isolates appear as “genetically independent” in the sense that all pairs exhibit more or less the same level of divergence. Clonal complexes are clearly visible in this tree and are represented by: 22 isolates belonging to CC-ST10; 2 isolates belonging to CC-ST90 (IT02 and FRGDSA 1882/11) which is another clonal complex known to infect rainbow trout in France, Switzerland and Chile (Siekoula-Nguedia et al., 2012; Strepparava et al., 2013; Avendaño-Herrera et al., 2014); and 2 isolates (NCIMB1947^T^ and OSU THCO2-90) belonging to CC-ST9 known to infect coho salmon in North America, Japan and Chile (Nicolas et al., 2008; Fujiwara-Nagata et al., 2013; Avendaño-Herrera et al., 2014; Van Vliet et al., 2016). In total 18 groups of isolates without obvious relationships between them could be distinguished, except for some weak proximity between CC-ST90 and CC-ST10 and maybe some moderate level of geographical population structure reflected by one group of three isolates of European origin (FI146, JIP 16/00, and DK095). A star-like tree could arise from a rapid population expansion at some point in the past near the root. However, it could also result from high amounts of recombination blurring the signal of the organismal genealogy. High amounts of recombination in the *F. psychrophilum* core genome were already detected based on MLST data, in particular via the level of apparent homoplasy (Nicolas et al., 2008). Here, we computed that to generate the genotypes of the 41 isolates containing 15,543 SNPs on a single tree it would require at least 43,009 nucleotide changes (value obtained for the most parsimonious tree found by dnapars). This corresponds to a Homoplasy Index (HI) of 64.2% [(43,009-15,418)/43,009] which is orders of magnitude higher than the amount of true homoplasy expected at these levels of sequence divergence. This result strongly corroborates the idea that the star-like shape of the tree reflects the homogenizing force of recombination.

In order to get additional clues on the evolutionary forces acting on the nucleotide diversity we also inspected the distribution of the Minor Allele Frequency (MAF). This was done after taking only one representative genome per CC to avoid arbitrary over-representation of some genotypes. The MAF spectrum built on all sites and represented in Figure 1C deviates only slightly from the theoretical prediction of the standard coalescent with the infinitely many sites mutation model (panmictic population, stable population size, low number of mutations, no selection). Strikingly, the match is almost perfect when considering only synonymous SNPs. In contrast, sites of non-synonymous polymorphism show a clear excess of low MAF. Tajima's D statistic, whose deviation with respect to 0 captures the amplitude and direction of the difference between the observed MAF spectrum and the neutral prediction (Tajima, 1989; Achaz, 2009), was −0.702 for non-synonymous sites but only −0.0027 for synonymous sites. Altogether the star-like shape of the tree, the high level of apparent homoplasy, and the excellent fit between the MAF of the synonymous sites and the standard neutral model suggest a highly recombinogenic and almost panmictic population with a relatively stable population size in the time frame of the species genealogy.

### Core-genome and pan-genome

Among the 4,805 gene families, 890 corresponded to short CDSs (maximum length in cluster < 100 aa). These were discarded from our comparative analyses of the gene content since short CDSs are typically more prone to be missed in annotation or to correspond to over-predictions or pseudogenes. In support of the use of this criteria, we computed that only 9.6% (85/890) of the families with short CDSs had at least one representative in each genome (core genome genes) whereas this fraction was as high as 43.7% (1,711/3,915) for families with longer genes.

We examined more systematically the distribution of the 3,915 gene families with CDSs longer than 100 aa. Each individual genome contained an average of 2,184 of these gene families. The core genome defined as the genes common to the 41 isolates consisted of 1,711 gene families and therefore accounted for 43.7% of all gene families. In an individual genome, the gene families decomposed approximately into 78.3% (1,711/2,184) for the core genome and 21.7% for the variable gene pool.

**Figure 2:**
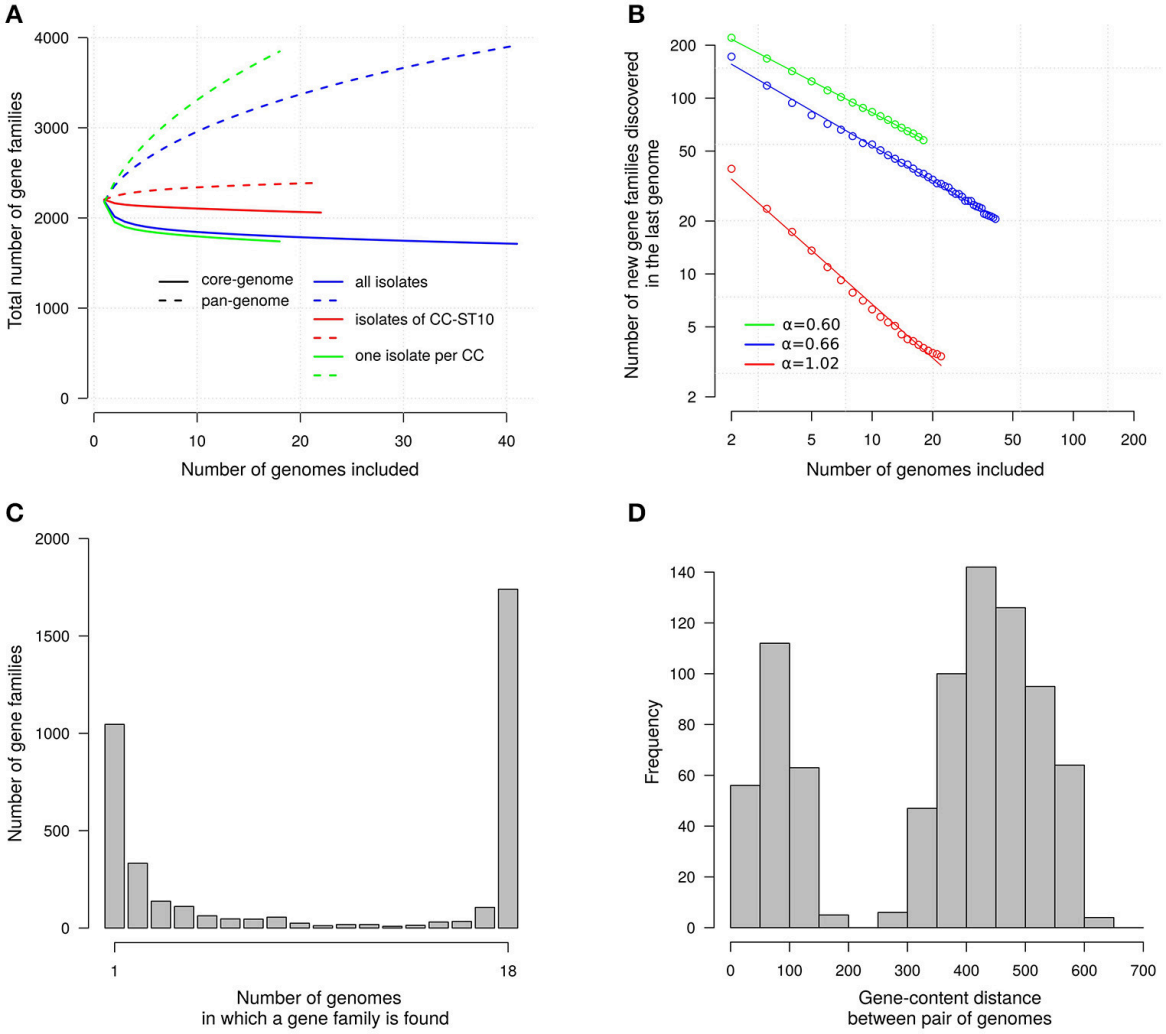
Gene families. **(A)** Accumulation curves for the sizes of the core- and pan-genomes. Core-genome size, number of gene families in common; Pan-genome size, total number of gene families. Number averaged on 10,000 randomized orders for genome addition. Only gene families of which one member has a length above 100 aa are represented. Colors distinguish curves for three sets of isolates: the 41 isolates considered in this study (blue), the 18 isolates obtained when taking only one representative per clonal complex (green), and the 22 isolates of CC-ST10 (red). **(B)** Fit of the power-law on the number of new gene families discovered in the last genome added; α is the scaling exponent corresponding to the slope in this log-log representation. Same colors as in **(A)**. **(C)** Gene frequency spectrum. Only one representative per CC is considered. **(D)** Distribution of gene-content distances between pairs of genomes.

To get insights on what could be the properties of the total gene repertoire of the species, defined as the pan-genome, we examined how the number of gene families increases when genomes are added one-by-one (averaging over 10,000 random orders for the additions). The resulting accumulation curve is shown in Figure 2A and clearly indicates that additional gene families will still be found when more genomes will be analyzed. However, the number of gene families will not increase rapidly. Indeed, when considering our set of 41 genomes the last genome added brings on average ~21 new gene families. This number does not account for the genetic structure of our sample where many isolates (22) belong to a single clonal complex. When considering 18 “genetically independent” isolates, obtained by taking only one representative isolate per clonal complex, the last genome brings ~58 new gene families. In sharp contrast, for the 22 isolates of CC-ST10 the last isolates brings only ~3.4 new gene families. We estimated the scaling exponent of the power law fitting the number of new genes discovered as a function of the number of genomes to: α = 0.66 when taking the 41 isolates; α = 0.60 when taking only one representative per clonal complex; and α = 1.02 when considering only the 22 isolates belonging to CC-ST10 (Figure 2B). Values below 1 for α are indicative of open pan genomes that are predicted to diverge as more genomes are sequenced (Tettelin et al., 2008).

To characterize more fully the distribution of the genes we also represented the frequency spectrum of the gene families (Figure 2C). It appears that most gene families in the variable gene pool (2,204 gene families) occur only in a small fraction of “genetically independent” isolates (63.4% in one or two isolates out of 18). The distribution of pairwise differences in gene-content between isolates also help to describe isolate-to-isolate variations (Figure 2D). This distribution is clearly bimodal. On the one side we find differences between pairs of “genetically independent” isolates which fall typically between 400 and 500 genes. On the other side the differences between pairs of isolates in a same clonal complex tend to be less than 100 genes. We decided not to display a tree reconstructed on the basis of gene content distances since our attempts indicated that the result was prone to contain artifacts reflecting the number of contigs.

### Whole genome MLST

For each multiple sequence alignment of CDSs, we analyzed the number and distribution of the allele-types (ATs). This approach, which summarizes SNP information at the locus-level and referred to as whole-genome MLST, is particularly relevant to investigate the diversification of clonal complexes in bacterial species with significant amount of recombination (Jolley and Maiden, 2010). Indeed, each recombination tract can incorporate several genetically linked nucleotide differences whose exact number is not relevant with respect to the reconstruction of the genealogical relationship between isolates. In this context, counting individual nucleotide changes does not properly account for the number of independent evolutionary events, increases variance of estimated branch lengths, and tends to give more weight to recombination than to mutation events.

**Figure 3:**
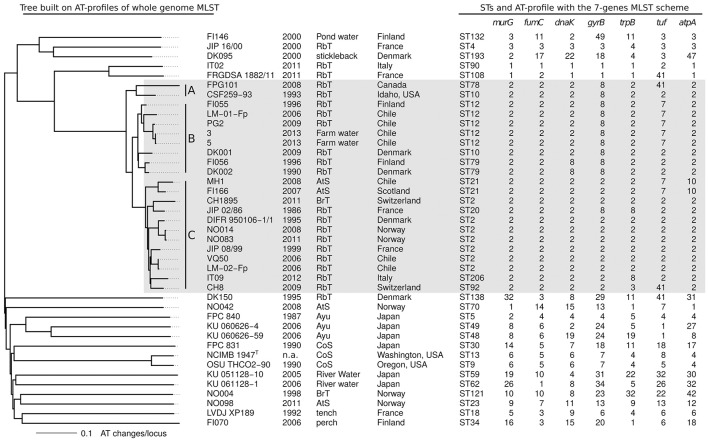
Whole-genome MLST. Neighbor-Joining tree built on pairwise distance between AT-profiles. Distance counted as the fraction of allele types that differs between two strains. Isolate name, year, host fish, and country of sampling are reported. The following abbreviations are used for the fish names: RbT for rainbow trout, CoS for coho salmon, AtS for Atlantic salmon, and BrT for brown trout. STs and AT-profiles corresponding to the classical 7-genes MLST scheme are also shown. CC-ST10 is highlighted by a light gray box.

For the 1,549 conserved single copy genes, the number of ATs per locus varies from 1 to 26 with a mean of 9.93. Pairwise differences between isolates were computed and a tree was built with the Neighbor-Joining approach to represent this information. This tree based on AT-divergence, shown in Figure 3, has a star-like shape similar to the tree built on nucleotide divergence (Figure 1B) but the details of the topology differ. The average AT-divergence for pairs of “genetically independent” isolates is around 0.8 AT changes per locus whereas two isolates from a same clonal complex exhibit typically less than 0.2 AT changes per locus. None of the pairs exhibited distance between 0.3 and 0.5, and consequently any cut-off in this range could serve to tell if two isolates are inside a same clonal complex or not. To facilitate integration with already published and future MLST data, we also superimposed on the whole genome MLST tree the STs and AT-profiles corresponding to the seven loci of the classical MLST scheme (Figure 3).

### Genetic diversity of CC-ST10

In CC-ST10, the tree built on AT-divergence distinguishes three groups of isolates: group A consists of the two North American isolates (CSF259-93 and FPG101), group B consists of eight isolates of North European and Chilean origins (from FI055 to DK002 when arranged in the topological order of the AT-divergence tree used Figure 3), and group C consists of 12 isolates of pan-European and Chilean origins (from MH1 to CH8 in Figure 3). Interestingly, pairs of closely related isolates sampled years apart tended to be at different distances from their most recent common ancestor (MRCA): FPG101 isolated 15 years after CSF259-93 is more distant of 0.059 AT changes per locus (distance to the MRCA of FPG101 and CSF259-93); CH1895 isolated 25 years after JIP 02/86 is more distant of 0.026; LM-01-Fp isolated 10 years after FI055 is more distant of 0.026. This was however not systematic since for instance DK001 isolated 19 years after DK002 is only more distant of 0.008. Furthermore, group B is globally at a closer evolutionary distance from the MRCA of CC-ST10 than isolates of group C.

The definition of groups A, B, and C is relevant to describe the pattern of AT-divergence in CC-ST10 but it is unclear if they all really represent distinct clades in the genealogy of this clonal complex. In particular, group B did not appear as monophyletic in the trees reconstructed by Neighbor-Joining or parsimony on CC-ST10 isolates only (Supplementary Figure 2). However the level of bootstrap support for the concerned branches is such that it does not allow a conclusion on this point. Parsimony reconstruction also permitted to measure the amount of apparent homoplasy inside CC-ST10 at the nucleotide [HI 24.9% which corresponds to (3,211-2,413)/3,211] and AT [HI 12.1% which corresponds to (1,290-1,134)/1,290] levels. These moderate amounts of apparent homoplasy indicate that even if recombination is the primary driving force of diversification within CC-ST10, it does not totally disrupt the genealogical signal. Of note, AT-profiles at the seven loci of the classical MLST scheme (Figure 3) exhibited on this tree a higher amount of apparent homoplasy than average conserved genes [HI 36.4% which corresponds to (11-7)/11].

In terms of gene content, the 22 isolates of CC-ST10 contained 88 conserved gene families (out of which 75 are ≥ 100 aa) not found in any of the 19 other isolates. These genes specific to CC-ST10 are listed in Supplementary Table 2. The vast majority (78/88) are located in three large genomic clusters flanked by repeated regions (i.e., tRNA or transposon elements). The first genomic cluster (FPSM_00422 to FPSM_00430, ~11 Kbp) contained genes putatively involved in DNA replication and repair. The second and largest genomic cluster (~67 Kbp) contained phage-related proteins, 8 putative transcription regulators (including a XRE family transcriptional regulator, FPSM_01245) and a restriction–modification system. We also noticed in this cluster the presence of the gene *tetX* (FPSM_01273) originally characterized in *Bacteroides fragilis* transposons (Speer et al., 1991) and encoding a flavin-dependent monooxygenase conferring resistance to tetracycline antibiotics by enzymatic degradation (Moore et al., 2005). Such a resistance mechanism might have provided a selective advantage to CC-ST10 in the context of rainbow trout farms, which have experienced recurrent antibiotic treatments over the past four decades.

The third genomic cluster of genes specific to CC-ST10 isolates (~35 Kbp) contained homologs to genes encoding sub-units of the Type II secretory apparatus (T2S) involved in protein translocation across the outer membrane in several pathogenic and non-pathogenic Gram-negative bacteria (Korotkov et al., 2012). The main structural components were identified, including: GspD (FPSM_02420), the secretin that provides the pore through the outer membrane; GspE (FPSM_02421), the cytoplasmic ATPase energizing the system; homologs of the inner membrane platform proteins GspC, GspL, and GspM; and GspG (FPSM_02423), the main periplasm-spanning pseudopilin acting as a piston. These T2S structural genes are organized in two loci transcribed in the opposite direction (FPSM_02418 to FPSM_02423 and FPSM_02435 to FPSM_02439). Several genes encoding exported proteins of unknown function were also identified. Some might participate to virulence, as exemplified by FPSM_02426 and FPSM_02429 which encode proteins with structural domains found in the ABC toxin of *Yersinia entomophaga* (Busby et al., 2013), or FPSM_02432 encoding a putative adhesin. The presence within this cluster of FPSM_02424, predicted to encode a member of the family OmpH/Skf of molecular chaperones which are known to interact with unfolded proteins as they emerge in the periplasm from the Sec translocation machinery (Walton and Sousa, 2004), reinforces the hypothesis of a dedicated role in protein translocation.

**Figure 4:**
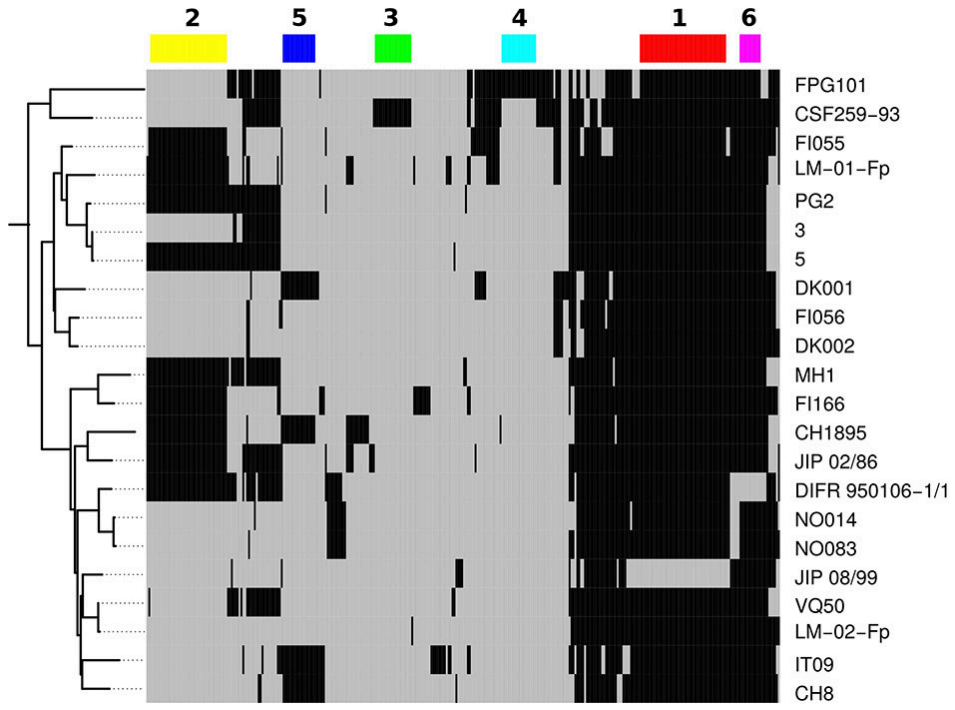
Variable gene pool of CC-ST10. A total of 330 gene families are represented here. Black indicates presence and gray absence in a particular genome. Genomes are ordered vertically according to the tree built on AT-profiles. Genes are arranged horizontally according to their presence/absence profile as obtained by hierarchical clustering based on Manhattan distance and complete link (not shown). The six major profiles are represented by color rectangles and numbered.

Only 330 gene families (≥ 100 aa) were not conserved among the 22 isolates of CC-ST10. In Figure 4 we display the distribution of this variable gene pool. When grouping gene families by distribution profile, six groups of more than 10 gene families were identified (numbered 1 to 6 in Figure 4). Taken together, these six groups accounted for 150 gene families. Group 1, consisted of 45 genes and seems to be an artifact since it distinguishes genes present in all CC-ST10 genomes excepted JIP 08/99 which has by far the highest number of contigs among these genomes. In contrast to the five other groups, and consistent with the interpretation as an artifact, genes in Group 1 were scattered over the genome (using completely assembled genomes such as CSF259-93 as a reference). Group 2 consisted of 40 gene families and corresponds to prophage 6H (Castillo et al., 2014) which is present in ~40% (9/22) of our CC-ST10 isolates. Groups 3, 4, and 5 accounted respectively for 19, 18, and 17 gene families and corresponded to probable mobile genetic elements found in one (groups 3 and 4) to four (group 5) isolates. They also contained several restriction/modification systems (groups 3 and 4). Group 5 contained genes encoding a transposase and proteins of unknown function and likely corresponds to a genomic island originating from a prophage bordered by a tRNA asn (FPDK001_tRNA22). Group 6 contained 11 genes organized into two contiguous stretches (8 genes FP0255 to FP0263 among which 3 are involved in L-homocysteine biosynthesis, and 3 genes FP2532, FP2531, and FP0627 encoding a hypothetical protein, a phage integrase and a transposase, respectively) that are lost in a single isolate (DIFR 950106-1/1). Interestingly, we note that the presence/absence profiles corresponding to groups 2 or 5 cannot be explained by a single event of gain or loss along one of the trees reconstructed on the basis of nucleotide sequences or AT-profiles.

**Figure 5:**
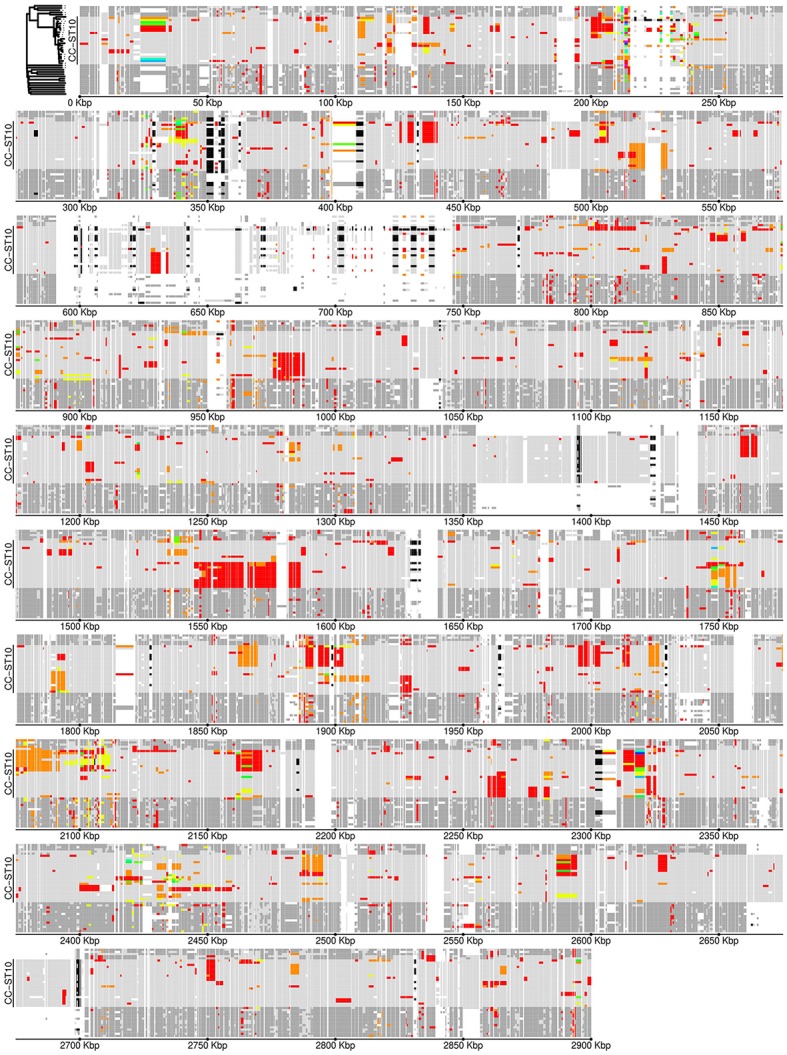
Allele-type map of CC-ST10. Protein coding gene loci are positioned along the genome according to gene coordinates in the genome of strain CSF259-93. Genomes are ordered vertically according to the tree built on AT-profiles as illustrated in the upper-left corner; the exact position of CC-ST10 is reported. Black indicates presence of a gene in more than one copy; white indicates absence. Allele-types in CC-ST10 are represented by different colors: light gray for the main allele-type and red to yellow for the others, with some local reallocations of colors to maximize the consistency of the color choice between adjacent loci. Allele-types not found in CC-ST10 are represented in dark gray.

To get a picture of the genetic organization of the AT-changes along the genome we drew a genome-wide map of the distributions of AT in CC-ST10 (Figure 5) showing all protein genes of CSF259-93, and not only the conserved single copy genes used for tree reconstruction. This map allows visualization of recombination tracts that introduce AT-level polymorphism extending over several contiguous genes. The recombination tracts visible on this map are those incorporating alleles from outside CC-ST10. Their lengths are heterogeneous: the longest could reach up to ~40 Kbp such as around position 1,550 Kbp but many extend only on a couple of genes. Qualitatively, it is clear that a large number of recombination events contributed to the genetic diversification within CC-ST10.

### Roles of recombination and mutation in the diversification of clonal complexes

**Figure 6:**
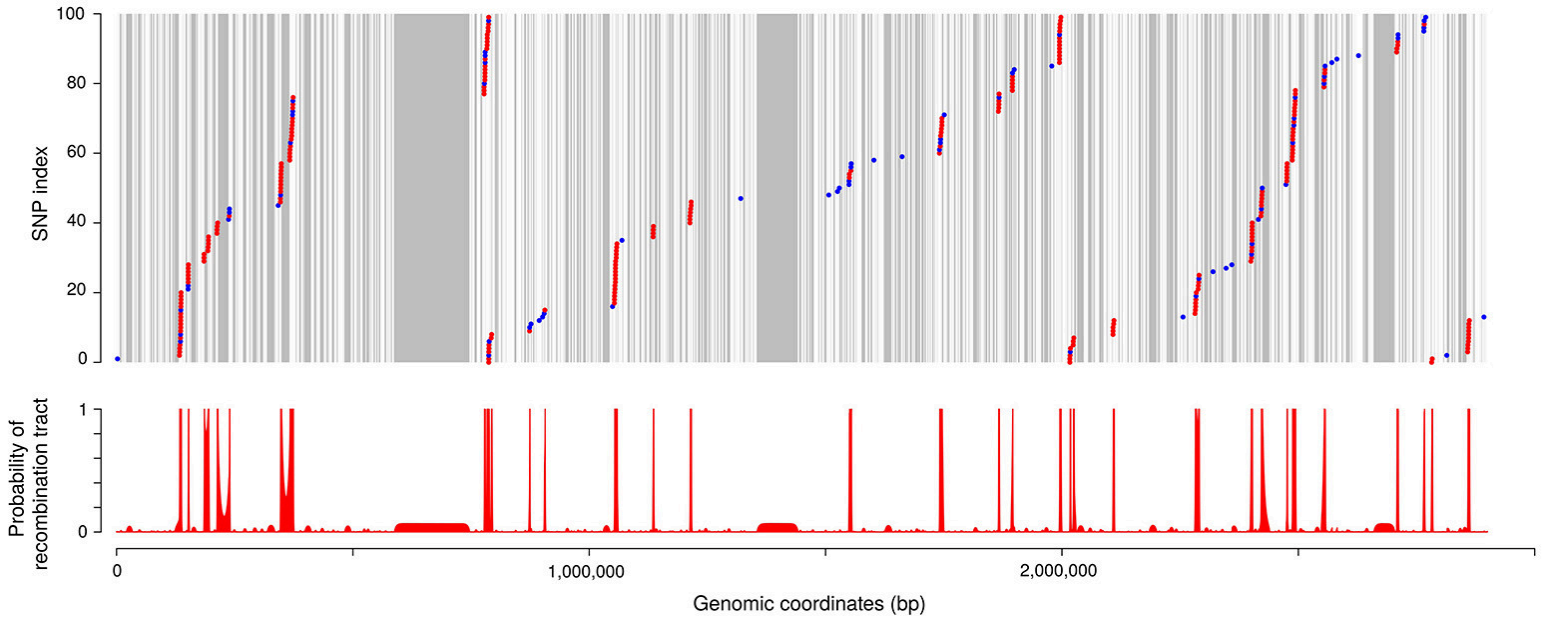
SNPs and recombination tracts between two closely related isolates. The strains compared here are JIP 02/86 and CH1895. **(Upper)** Positions of the SNPs along the genome (using the genomic coordinates of CSF259-93 as references). SNP index is reset every 100 SNPs for this representation. Each dot represents one SNP between the two isolates. Colors distinguish two types of polymorphism: in blue, polymorphism observed only inside CC-ST10 (referred to as polymorphism of type P1 in the text); in red, polymorphism observed also outside of CC-ST10 (polymorphism of type P2). Areas in gray correspond to regions not covered by our alignments. **(Lower)** Probability of recombination tract as computed with the HMM.

Disentangling the respective roles of recombination and mutation in the diversification of clonal complexes would allow to assess quantitatively the contributions of these two evolutionary processes and to analyze much more precisely their respective properties. For this purpose we examined, at the nucleotide level, the pairwise divergence between closely related isolates along the genome. We also distinguished two types of polymorphism (P1 and P2) according to whether one allele was specific to the clonal complex considered as expected from a mutation event (P1) or if both alleles were also found outside of the clonal complex as one would expect when many nucleotide changes were introduced by recombination (P2). A typical organization of the SNPs between two isolates examined from these perspectives is shown in Figure 6. Visual examination confirmed the existence of defined regions with high P2 and moderate P1 corresponding to recombination tracts interspersed in a background of low P1 and no P2 corresponding to non-recombined regions. A HMM was used to automatically delineate recombination tracts based on local rates of P1 and P2. The results are illustrated on Figure 6 (see Materials and Methods for details on the methodology).

**Figure 7:**
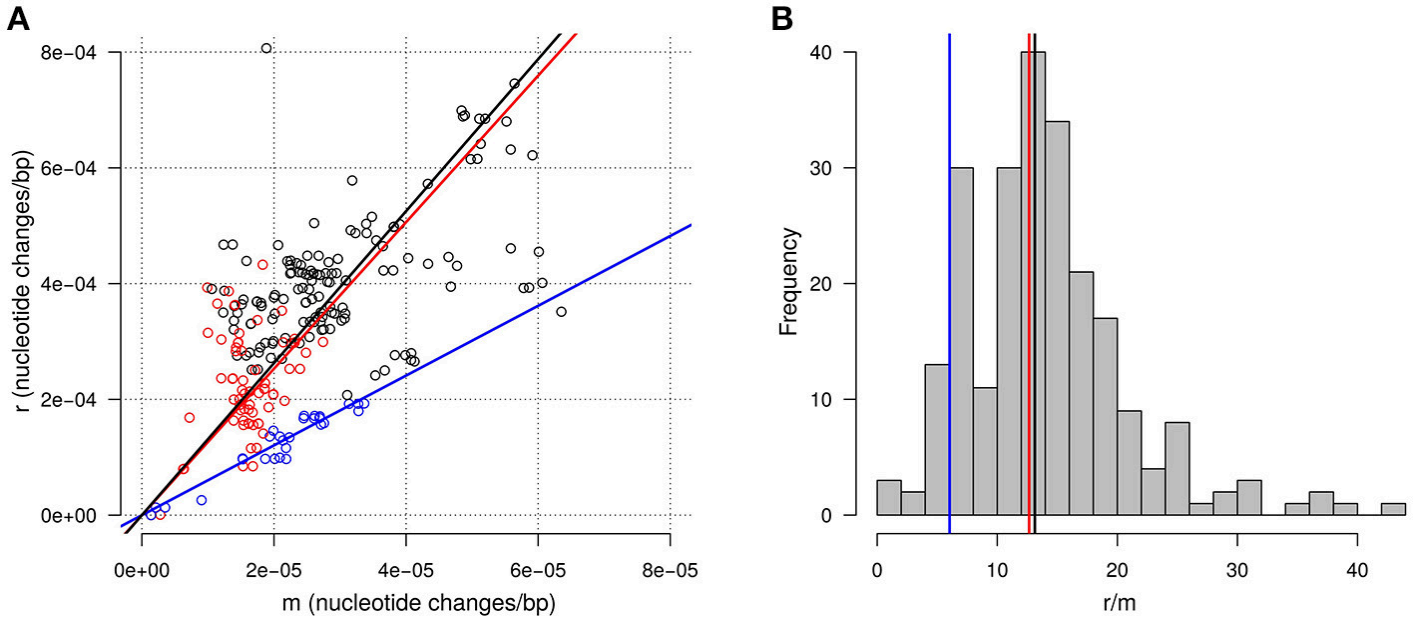
Recombination and mutation in divergence between closely related isolates. **(A)** Relationship between the amount of nucleotide changes arising from recombination (r) and from mutation (m). Each point represents a pair of closely related isolates. Colors highlight pairs within two groups of isolates in CC-ST10 according to the topology of the tree built on AT-profiles: blue for isolates from FI055 to DK002 (group B in the text) and red for isolates from MH1 to CH8 (group C in the text). The lines represent the line with intercept zero and slopes equal to the median of r/m for all pairs (black), for pairs in group B (blue), and for pairs in group C (red). **(B)** Distribution of the estimated r/m ratio across pairs of isolates. Vertical lines correspond to the slopes of the lines in **(A)**.

The HMM approach which assumes a geometrically distributed length for the recombination tracts allowed us to estimate more precisely the average length of the recombination tracts to 4.0 Kbp. Importantly, estimation with the HMM of the frequency of P1 in the non-recombined background also provided an estimate of the divergence caused by mutations between each pair of isolates. Reciprocally, the divergence caused by recombination was estimated by summing the estimated probability of being in a recombination tract at each polymorphic position and dividing this value by the cumulated length of the alignments considered (1,489 Kbp distributed into 1,500 single copy genes). Figure 7A shows how these two components of the divergence, hereafter referred to as m for mutation and r for recombination, relate to each other across all pairs of isolates considered. As expected for two evolutionary distances, a clear positive correlation is visible between r and m (Pearson correlation coefficient 0.61). The median of the distribution of r/m is 13.1 indicating that, on the time-scale of the diversification of the clonal complexes, the contribution of recombination to nucleotide changes is one order of magnitude greater than the contribution of mutation.

The variability of the ratio r/m is also noticeable with an interval between the 5 and the 95% quantile spanning 5.5 to 25.8 (Figure 7B). This variability probably reflects in large part the inherent randomness of the number underlying biological events. However, a fraction of this variability might also well be linked to biological or environmental differences between lineages. For instance, we noticed that pairs of isolates belonging to group B, as defined on the basis of the tree built on AT-profiles, tend to exhibit a lower r/m with a median 6.0 (Figures 7A,B). Also consistent with the idea that r/m could vary between lineages, the highest r/m ratio (42.8) was obtained for the pair of isolates belonging to CC-ST90 (FRGDSA 1882/11 and IT02). Nevertheless, differences between lineages were not systematic since the r/m for the pair of isolates belonging to the clonal complex CC-ST9 infecting coho salmon (OSU THC02/90 and NCIMB1947^T^) was estimated at 15.9 and thus appeared well in the range of the values obtained in CC-ST10 infecting rainbow trout.

Mutation contributes less than recombination to nucleotide sequence divergence within clonal complexes but we noticed that it introduces a different type of changes leading often to non-synonymous polymorphism. In CC-ST10, 849 positions out of a total of 2,413 in the 1,500 conserved single copy genes were found to harbor non-synonymous polymorphism. This represented ~35.2% of non-synonymous polymorphism, mainly introduced by recombination as estimated via the ratio r/m. However, if we consider the 144 positions that are outside all detected recombination tracts (probability ≤ 0.25 across all pairs of isolates) this ratio raises to 72.9% (105/144). This observation suggests that mutations arising in the short time frame of clonal complex diversification are not all typical of those reaching high allele frequency in the population (the bulk of the differences between more distant isolates). Indeed, it is tempting to speculate that many of the non-synonymous mutations detected in clonal complex diversification may not be neutral and therefore may not reach high allele frequency. This idea is qualitatively consistent with our observation that non-synonymous polymorphisms between “genetically independent” isolates tend to display lower MAF than synonymous polymorphisms (Figure 1C).

### Mutational distances in CC-ST10

**Figure 8:**
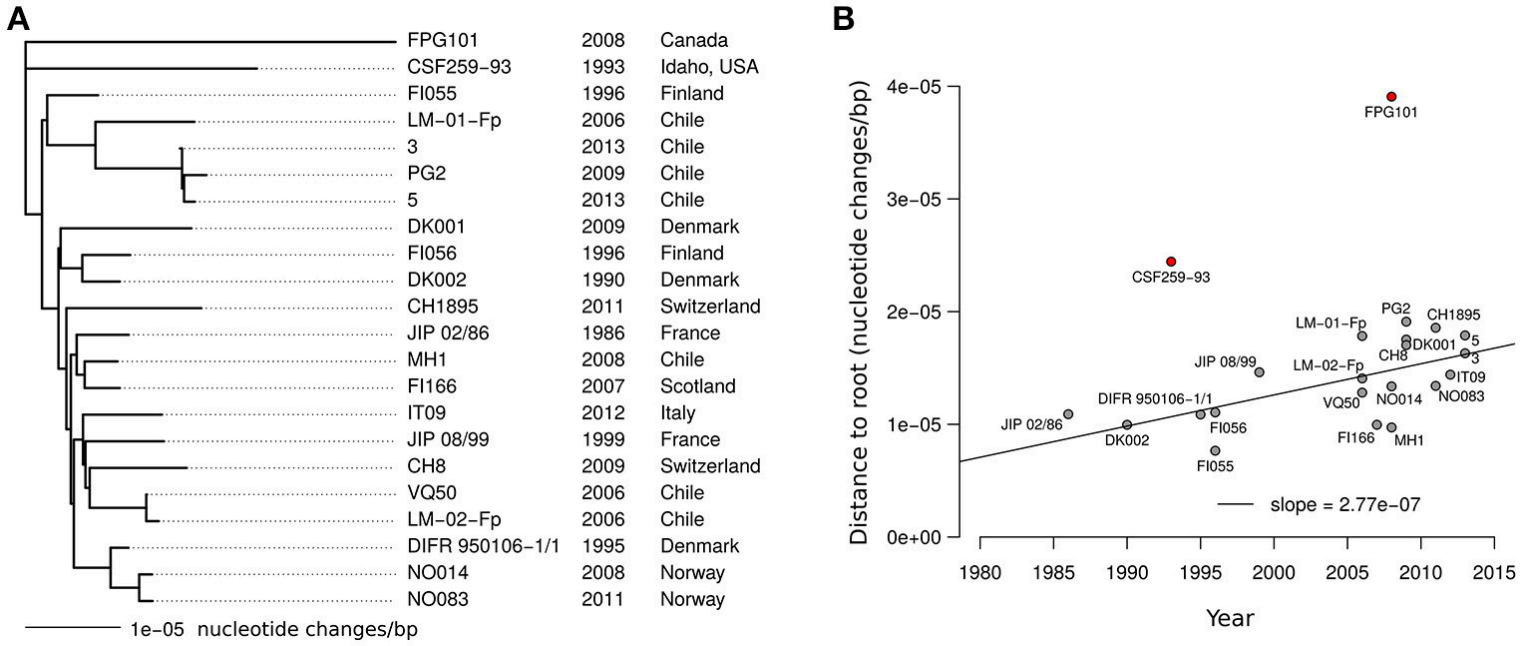
Tree built on mutational distances within CC-ST10. **(A)** Neighbor-Joining tree for CC-ST10 built on the estimated number of mutations (m). **(B)** Tip to root distance as a function of isolate sampling year. The line represents the linear regression obtained after excluding the two isolates of North-American origins (shown in red).

The most striking features of the pairwise estimates of m are probably the very short evolutionary distances at play. The maximal pairwise distance is found for the two North American isolates (CSF259-93 and FPG101) and amounts to 6.35e-5 nucleotide changes/bp. Among the 20 isolates sampled outside North America the maximal distance is only 3.55e-5 nucleotide changes/bp (between CH1895 and PG2). We used the estimated pairwise values of m as an evolutionary distance to build a picture of the genetic relationships between the 22 CC-ST10 isolates that would account only for nucleotide changes resulting from mutation events during the diversification of the clonal complex. The tree obtained by Neighbor-Joining is show in Figure 8A. In terms of topology, the tree is slightly different from the global tree built on AT profiles shown in Figure 3 but very similar to those reconstructed on CC-ST10 (Supplementary Figure 2). However, there is clearly little signal to reconstruct the exact topology from m only as indicated by the short internal branches.

Since the approach formalized in the HMM would only detect recombination tracts incorporating genetic material from outside CC-ST10 but not recombination that would occur between lineages belonging to CC-ST10 we were also interested in assessing the level of phylogenetic incongruence. For this purpose, we computed the amount of apparent homoplasy by applying the parsimony method to the 144 positions outside recombination tracts (Supplementary Figure 3). The most parsimonious trees required only 147 changes corresponding to an HI of only 2.0%. This polymorphism is therefore globally consistent with little recombination. However, the short internal branches in this tree indicate that most SNPs are not topologically informative and thus the interpretation of the low apparent homoplasy as an absence of recombination should be taken very cautiously. Indeed, recombination between lineages of a population with such a genealogy may incorporate changes produced by mutations during CC-ST10 diversification on lineages not included in our sample and this would not produce phylogenetic incongruence.

Information about the root of CC-ST10 genealogy was also obtained using parsimony tree reconstruction applied on the 144 SNPs outside of any recombination tracts. Here, we took advantage of the knowledge of the ancestral allele for these SNPs which are all of type P1 and have thus only one allele found outside CC-ST10 (almost certainly the ancestral allele). By including the ancestral genotype composed of these alleles for the 144 SNP positions in the phylogenetic reconstruction (Supplementary Figure 3) we placed the root of CC-ST10 near the meeting point of the three branches connecting isolate CSF259-93 to isolate FPG101 and to the other isolates, as represented in Figure 8A.

Interestingly, in the tree based on mutational distance (Figure 8A), the most recent isolates tend to be farther from the root. Positive correlation was detected between distance to the root and year of isolation using rank-based measure of correlation (Spearman rho = 0.45, *p*-value = 0.035). However, Pearson correlation, which is more sensitive to outliers, was not statistically significant due to greater distances to the root for the two North American isolates than for other isolates (Figure 8B). When considering the 20 isolates sampled outside North America, Pearson correlation was statistically significant (Pearson *r* = 0.64, *p*-value = 0.0024). At least two ingredients could contribute to the distinct behavior of the North American isolates. The first is a better positioning of the root of the subtree corresponding to the other 20 isolates. The second is a higher rate of molecular evolution for North American lineages which may possibly reflect higher amount of nucleotide changes incorporated by recombination events with other lineages of CC-ST10 (not detected by the HMM) as expected for instance if the diversity within CC-ST10 is greater in North America. A rough estimate of the rate of molecular evolution per year was obtained by fitting a linear model on the 20 isolates from outside North America and led to 2.77e-7 nucleotide changes/bp per year (s.d. 7.86e-8) as shown in Figure 8B.

### Plasmid repertoires

The presence of plasmids among *F. psychrophilum* isolates was noticed since the earliest genetic studies and plasmid profiles have been analyzed for correlations with variations in virulence, host fish, geographic origin, serotype, genotype, antimicrobial susceptibility, and for epidemiological purposes (Cipriano et al., 1996; Chakroun et al., 1998; Madsen and Dalsgaard, 2000; Kim et al., 2010; Henríquez-Núñez et al., 2012; Ngo et al., 2017). To date, only pCP1 (CP for *Cytophaga psychrophila*, a deprecated name for *F. psychrophilum*) which is a 3,407 bp-long cryptic plasmid isolated from *F. psychrophilum* D12, has been sequenced to completion and proved to be very useful for the development of genetic tools (McBride and Kempf, 1996; Alvarez et al., 2004).

**Figure 9:**
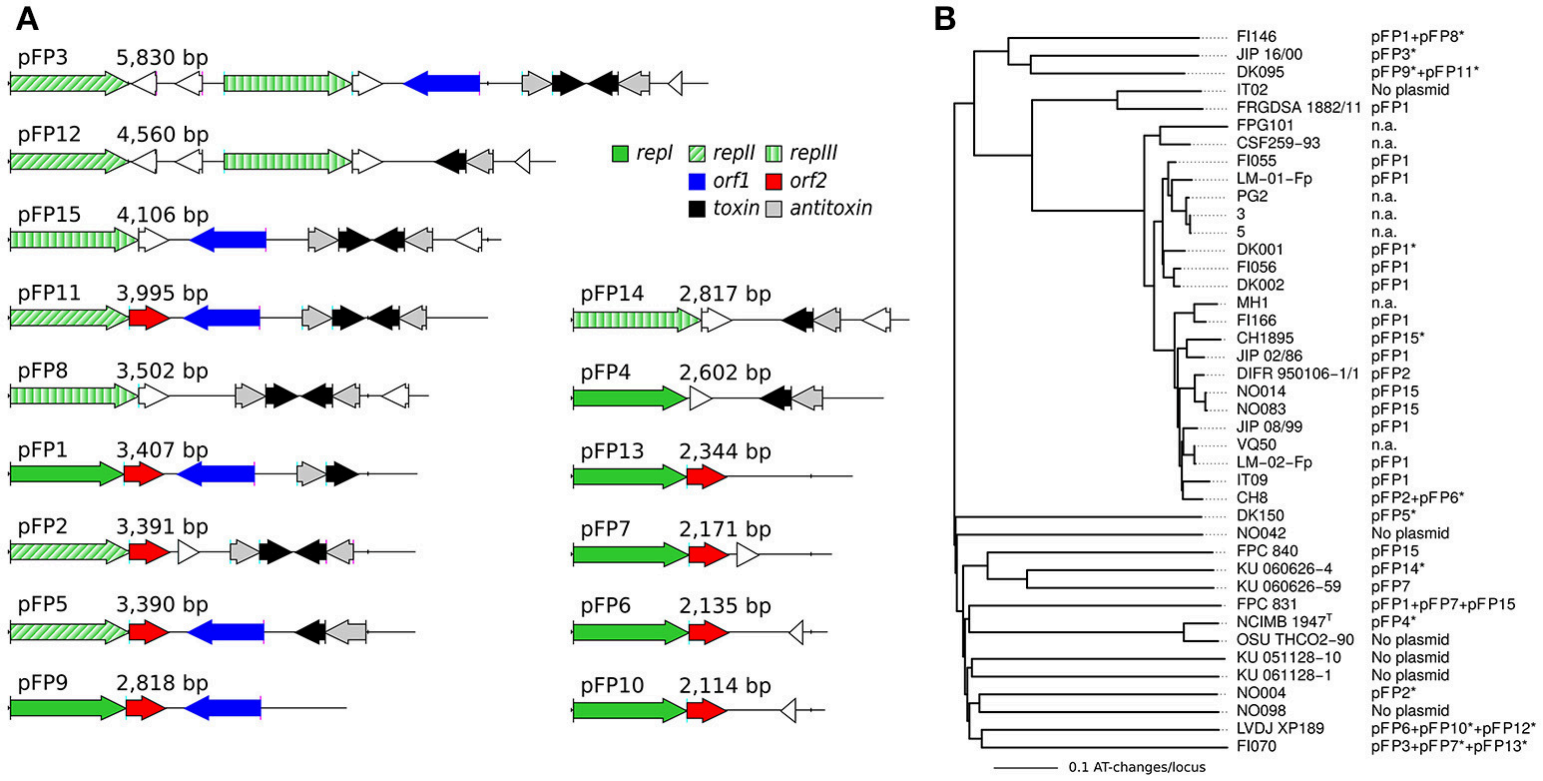
Plasmids found in our collection of isolates. **(A)** Physical maps of the 15 types of plasmids (pFP1 to pFP15) are ordered by length. Colors indicate the main different types of genes (see inset legend), pseudogenes, and unknown short genes are represented in white. Three types of *rep* genes have been distinguished based on the important divergence of amino-acid sequences. **(B)** Distribution of the 15 types of plasmids across the isolates. The Neighbor-Joining tree obtained on whole-genome MLST profiles is used here. n.a. stands for no data available. Asterisks (^*^) indicate the representatives that served to illustrate the different types of plasmids.

Physical maps of the 15 types of plasmids found in our genome sequences are shown in Figure 9A and their distribution across isolates is represented in Figure 9B. These plasmids are hereafter referred to as pFP1 to pFP15 since names pCP10 and pCP11 were already in use for two derivatives of pCP1 (McBride and Kempf, 1996). pFP1 which corresponds to pCP1 was identified in 13 isolates. Strikingly, most of the isolates contained at least one plasmid (only four isolates were devoid of plasmid). Furthermore, we found that there exist numerous plasmid types of about the same size and, as reported in Chakroun et al. (1998), some isolates harbor as much as three different plasmids.

All plasmid sequences contained at least one gene encoding a replicase. Three types of replicase encoding genes diverging widely by their amino-acids sequences (amino-acid identity ≤30%) were identified (*repI, repII*, and *repIII*) and some plasmids (i.e., pFP3 and pFP12) contained two types of replicase encoding genes. Most of the plasmids (10) contained an addiction module (i.e., toxin/antitoxin system) thought to be involved in plasmid maintenance. These genes could be on the leading strand such as in pFP1, on the lagging strand such as in pFP5, or even duplicated and positioned head-to-head such as in pFP15. The latter plasmid seems to be a truncated version of pFP3 (their sequences are identical but pFP15 lacks the 5' region of pFP3). The two other genes identified, orf1 and orf2, previously reported in pFP1 by Alvarez et al. (2004), are not always found.

## Discussion

The goal of this study was to draw a global picture of the genomic diversity of the *F. psychrophilum* species and to obtain insights on the evolutionary processes that shape this diversity at different time-scales. For this purpose, we built a collection of genomes encompassing both a set of isolates representative of the diversity of the species and a set of isolates belonging to a same clonal complex. Namely, 30 *F. psychrophilum* isolates were selected according to their MLST profile, geographic origin, and host fish species for complete genome sequencing. We also included fully assembled genomes for 11 isolates available from previous publications (Duchaud et al., 2007; Wiens et al., 2014; Wu et al., 2015; Castillo et al., 2016; Rochat et al., 2017a) but these are far from being random representatives of the *F. psychrophilum* diversity since they all belonged to only two clonal complexes.

The results largely corroborate the conclusions drawn from the initial analysis of MLST data (Nicolas et al., 2008). *F. psychrophilum* appears as a highly cohesive bacterial species characterized by genome sequences with low levels of nucleotide divergence (0.285% inside CDSs in pairwise sequence comparisons) when typical bacterial species can exhibit up to ~5% nucleotide divergence (Kim et al., 2014). This result is also in sharp contrast with the situation of *Flavobacterium columnare* for which three clearly distinct genomovars coexist (Kayansamruaj et al., 2017; Kumru et al., 2017). Another characteristic of the *F. psychrophilum* species is the very high amount of allele exchanges by homologous recombination that it exhibits at conserved loci. This is seen in the level of apparent homoplasy (HI 64.2%), which indicates that the distribution of polymorphism in the isolates is essentially incompatible with a tree whose topology would be common to all sites. In fact, due to the homogenizing effect of recombination, the footprints of the genealogical relationships between lineages vanish rapidly. Consequently, isolates that do not belong to a same clonal complex tend to be almost genetically independent as displayed in the star-like tree representing the genetic relationships within the species.

Importantly, the genome sequences allowed a precise estimation of the allele frequency spectrum of nucleotide polymorphisms within the species. Comparison of this spectrum with the theoretical prediction of the standard neutral model reveals an almost perfect match for the sites of synonymous polymorphism, as summarized by a value of the Tajima's D statistic close to 0. This suggests strongly that the low level of sequence divergence and the shape of the tree depicting genetic relationships is not the consequence of a recent expansion of the species since this standard model assumes a constant population size. This suggests also an absence of strong geographical population structure as the model assumes a panmictic population. The lack of population structure is in agreement with the absence of clear links between the topology of the tree built on nucleotide divergences and the geographical origin of the isolates. The negative value of D (−0.702) for sites of non-synonymous polymorphism is in line with the locus-level data compiled from many species which was interpreted as a result of ongoing purifying selection that probably prevents many of these polymorphisms from spreading through the population by genetic drift (Hughes, 2005). It is tempting to speculate that the high recombination rate may have played a role in the good fit of the allele frequency spectrum with the theoretical predictions of the standard neutral model. Recombination uncouples the fate of the neutral mutations and thus the allele frequency spectrum from the fate of the mutations under purifying and adaptive selections and from the organismal genealogy as illustrated in simulations of bacterial populations (Lapierre et al., 2016).

Genome sequences also allowed us to compare genome sizes and gene contents between isolates despite the inherent limitations of relying for these analyses on genomes that are not all completely assembled. The isolates harbor similar genome sizes around 2.7 Mbp and a standard deviation of only 0.1 Mbp. The total number of gene families that we detected in the 41 genomes is relatively limited (4,805) and the fraction of each genome accounted for by the variable gene pool in each genome is only ~22%. Nevertheless, the number of new genes discovered when new genomes are added decreases only slowly since most of these genes are found in a small number of genomes. Indeed, our results suggest an “open” pan-genome for the species, which means that the pan-genome is predicted to diverge (i.e., does not converge toward an upper-limit) when new genomes are sequenced (Tettelin et al., 2008). We estimated the scaling exponent (α) of the power law fitting the number of new genes discovered as a function of the number of genomes to ~0.60 while α ≤ 1 for an open pan-genome and α > 1 for a closed pan-genome. As a point of comparison this scaling exponent is lower than the nine examples reported in Tettelin et al. (2008).

Our data set includes 22 genomes belonging to CC-ST10 and two other genome pairs representative of two additional clonal complexes. This sampling scheme provides interesting raw material to assess the general characteristics of evolutionary dynamics on short evolutionary time scales and to analyze the genealogy of CC-ST10 which is found in rainbow trout farms worldwide. As a first step in this direction, whole genome MLST permitted a more detailed view of the genetic relationships within CC-ST10 since it distinguished isolates with the same ST in the 7-genes MLST scheme. The tree built on whole genome MLST profiles also suggested that ST10 (in the 7-genes MLST scheme) may be the ancestral ST (founder) of this clonal complex because it is found on both sides of the tree (with respect to the presumed position of the root). This hypothesis of ST10 as ancestral to this clonal complex was already proposed on the basis of its high number of SLV-links and lead to propose a renaming of CC-ST2 as CC-ST10 (Nilsen et al., 2014). Of note, ST10 has also frequently been found in rainbow trout farms in North America whereas ST2 has not yet been found in this region (Van Vliet et al., 2016). The prediction of ST10 as ancestral is thus consistent with the hypothesis of a dissemination of the clonal complex out of North America when rainbow trout have been introduced in other regions of the world.

A key aspect to understand evolutionary dynamics of clonal complexes is to disentangle the respective contribution of mutation and allele exchange by recombination to nucleotide divergence on these short evolutionary time scales. For this purpose, we employed a HMM to delineate regions of high SNP density between pairs of closely related strains. HMMs constitute a natural and well-established framework to model the effect of recombination tracts on polymorphism in bacteria. They have been used to analyze pairs of genotypes that terminate branches trying to depict the organismal genealogy (Didelot and Falush, 2007; Zhou et al., 2014; Didelot and Wilson, 2015) or to identify genetic exchanges between populations without referring directly to pairs of genotypes (Mostowy et al., 2017). Compared to these other HMMs, the main originality of our approach is to focus on pairs of genotypes and to distinguish two types of SNPs depending on whether the two alleles are also found elsewhere (implying apparent homoplasy). This idea generalizes an already described approach for analyzing SLVs in classical MLST (Feil et al., 2000). Another difference is that here we analyzed all pairs of closely related isolates (sharing more than 50% of the ATs in whole genome MLST) which remained manageable (231 pairs for CC-ST10, 1 pair for CC-ST9, and 1 pair for CC-ST90) and was easy to parallelize on a CPU cluster. SNPs of type P2 where found to be ~10 times more frequent than SNPs of type P1 in recombination tracts. In contrast, the low overall polymorphism of the species suggest that only a very small proportion of mutations occur at sites that are already polymorphic between distant isolates in our collection and thus generate polymorphism of type P2. A choice was made to consider that SNPs of type P2 could arise only in recombination tracts. This choice simplifies the likelihood landscape and maximizes the ability to detect SNPs incorporated by very short recombination tracts as identified in several species (Mostowy et al., 2014; Bubendorfer et al., 2016). Nevertheless, our approach estimated the average recombination tract length to 4.0 Kbp for *F. psychrophilum* which is relatively long. In the *Escherichia coli* species or the *Bacillus cereus* group average lengths substantially below 1 Kbp have been estimated (Didelot et al., 2010, 2012). However, longer recombination tracts were also reported, for instance in *Clostridium difficile* (He et al., 2010).

Maximum-likelihood estimation of the HMM parameters provided values for the ratio of the contributions of recombination and mutation (r/m) to nucleotide divergence between each pair of isolates. The results indicated a median r/m across pairs of 13.1 which is high. However, it is lower than our initial estimate of 26 obtained with the method of Feil et al. (2000) using MLST on 11 genes. It is also below the 95% credibility centered around 63.6 estimated on the subset of 7 more polymorphic genes selected for the *F. psychrophilum* MLST scheme (Vos and Didelot, 2009) using the approach of Didelot and Falush (2007). Interestingly, we noticed marked differences between the value of r/m in different phylogenetic sub-groups of isolates within CC-ST10. This heterogeneity might reflect differences in the frequency of co-infection between one isolate inside and one isolate outside of CC-ST10 or differences in intrinsic recombination rates.

We also noticed that SNPs outside recombination tracts correspond predominantly to non-synonymous changes (~72.9%) in sharp contrast with the general distribution of SNPs (~35.2% for the 22 isolates of CC-ST10; ~33.5% for the 41 isolates of the species). This high proportion is close to what is expected if mutations were distributed randomly on the genome. Namely, the fraction of non-synonymous sites on the genome is ~79% as obtained for N/(N+S) with the method implemented in yn00 (Yang and Nielsen, 2000). A very similar amount of non-synonynomous changes introduced by mutations in clonal complexes (~70% non-synonymous) were already reported in other bacterial species (Feil et al., 2003; Holden et al., 2004). More generally a negative relationship has been documented between the relative amount of non-synonymous vs. synonymous changes (summarized in dN/dS) and evolutionary distance (Rocha et al., 2006). The main explanation proposed is that a high proportion of mutations that do not spread in the population due to purifying selection are seen on these short time scales. This explanation is also in line with the negative Tajima D measured here on non-synonymous sites. However, an even higher dN/dS (>1) in long-term experimental evolution in *E. coli* was also identified and interpreted as a footprint of adaptive evolution (Wielgoss et al., 2011; Tenaillon et al., 2016). Indeed, part of the non-synonymous mutations detected within clonal complexes may correspond to (presumably local) adaptive evolution. However, inspection of the genes containing these mutations did not reveal clear accumulations of independent mutations in certain genes (not shown) and therefore did not provide clues on potential targets of adaptive evolution.

The afore-described HMM does not allow to detect recombination events between lineages of CC-ST10 but these presumably also contribute to the diversification of CC-ST10. The analysis of apparent homoplasy inside CC-ST10 at the ST (HI 12.1%) or nucleotide level (HI 24.9%) suggests that recombination between lineages of CC-ST10 also exists although homoplasy can be due to incorporation of alleles from outside CC-ST10 at a same locus in independent lineages. Importantly, recombination within the same genome is yet another source of genetic variation that contributes to diversification as shown for the chromosomal inversion detected when comparing two isolates of CC-ST10 (JIP 02/86 and CSF259-93). This large inversion may be recurrent in *F. psychrophilum* since it is bordered by conserved inverted repeats and it occurred on a short evolutionary time scale. However, its frequency and functional significance remains to be analyzed. For instance a chromosomal flip-flop system of *S. aureus* was found to be involved in a phenotypic switch between small and normal colony variants (Cui et al., 2012).

Estimation of pairwise mutational distance allowed us to reconstruct a tree by neighbor-joining distance that intends to depict more faithfully the genealogical relationships between isolates of CC-ST10. We were particularly interested in re-estimating evolutionary distances since the amount of recombination is very high in *F. psychrophilum* and the nucleotide changes caused by recombination accumulated apparently at different rates between lineages. Probably the most salient feature of the evolutionary tree built on these re-evaluated distances was its small depth; the more distant isolates were separated only by 6.35e-5 nucleotide changes per site. Having sequenced isolates collected over a period of 27 years, we detected a tendency for more recent isolates to appear in the tree as more distant to root, and we estimated the rate of molecular evolution at 2.77e-7 nucleotide changes/bp per year (s.d. 7.86e-8). This corresponds to changes introduced by mutations or recombination within CC-ST10 (outside North America) which could not be distinguished from mutations by the approach used to delineate recombination tracts. According to this value, the maximum divergence of 6.35e-5 measured in CC-ST10 (between CSF259-93 and FPG101, both isolated in North America) would correspond to ~229 years and thus, given the sampling date of the concerned isolates, a MRCA approximately in year 1886 [obtained as 1993-(229-15)/2]. For the 20 isolates sampled outside of North America the maximal pairwise distance is 3.55e-5 nucleotide changes/bp (between CH1895 and PG2) and would correspond to an MRCA for this group of isolates around year ~1946 [obtained as 2009-(128-2)/2]. The expansion and diversification of clonal complex CC-ST10 would thus have paralleled the development of rainbow trout farming during the last century, first in North America, whose Pacific coast is also the native range of rainbow trout, and then in the rest of the world. Such an expansion could also explain the star-like shape of the tree reconstructed for CC-ST10 on the basis of mutational distances (Figure 8A).

The calibration of the molecular clock at 2.77e-7 nucleotide changes/bp per year used to obtain these approximate times for the MRCAs does not seem unrealistically high. It can be compared to values reported for other bacteria in other contexts. In particular, the rate of mutation for several human pathogens has been estimated based on temporal genomic data at around 1e-6 per site and per year (Harris et al., 2010; Croucher et al., 2011; Mutreja et al., 2011; Hsu et al., 2015). As another point of comparison, mutation rates per generation have also been estimated *in vitro*. Based on experiments measuring mutation frequency at a specific-locus, Drake (1991) proposed a “universal” rate of spontaneous mutation of ~3e-3 per genome and per generation which would correspond to ~1.2e-9 per bp per generation for a genome the size of *F. psychrophilum*. Combined with an optimal generation time of 3h as measured in our lab for *F. psychrophilum* in TYES medium at 18°C, this would result in a rate of ~3.5e-6 nucleotide changes/bp and per year. This rate, which is one order of magnitude higher than our estimate, is a maximum in the sense that it does not take into account purifying selection which removes deleterious mutations, and the average generation time in the natural niche is probably higher than 3 h. In a mutation-accumulation set-up allowing for the accumulation of all but the most deleterious mutations, the spontaneous rate of mutation in *E. coli* was measured to ~2e-10 per bp per generation using genome sequencing (Lee et al., 2012). In a long term evolutionary experiment implying more purifying selection (Drake, 2012) this rate was measured to ~1e-10 per bp per generation (Wielgoss et al., 2011). Our estimated value of 2.77e-7 nucleotide changes/bp per year may for instance be obtained by rescaling the value of 1e-10 per bp for the genome size of *F. psychrophilum* and by combining it with a generation time of 6 h in the natural niche (i.e., substantially more than the 3 h in optimal laboratory conditions).

Beside the global picture of the genomic diversity and evolutionary processes in the species *F. psychrophilum* presented here, the newly sequenced genomes will probably prove useful to identify genes underlying variation in some interesting phenotypes. We have for instance used these data to identify genes determining differences in serotypes (Rochat et al., 2017b). Likewise, the presence of *tetX* and of genes encoding a type II secretion system among the genes that are specific to CC-ST10 suggests possible links with antibiotic resistance and virulence. Finally, the analysis of plasmid sequences indicates that gel electrophoresis migration profiles cannot distinguish all the different plasmids circulating in *F. psychrophilum*. Furthermore, the genes carried by these plasmids appear as a variation on a same theme with 5 genes encoding a replicase, Orf1, Orf2, a toxin and an antitoxin and seem thus unlikely to be relevant for key phenotypes such as virulence or antibiotic resistance. To date, only strain OSU THCO2/90 is permissive to genetic manipulations based on a pFP1 (pCP1) derivative, a characteristic likely due to the absence of plasmids in this strain. The detailed description of the different plasmids resulting from the present study, which for instance highlights the existence of different types of replicases, could help develop new genetic tools that may work in strains that already contain some plasmids.

## Author contributions

ED and PN were the primary investigators of this study. Their work encompassed the design of the study, the bulk of the *in silico* analyses, and the writing of the manuscript. TR, CH, PB, VL, CG, ID, LM, HN, KS, ToW, NS, ThW, GC, AM, GW, EF-N, RA-H, and J-FB helped with the design of the study and interpretation of the results and approved the final version of the manuscript. More specifically, J-FB contributed to the choice, cultivation and management of the isolates as well as the writing of the manuscript; CH assembled the genomes and conducted a pilot analysis of this dataset; and TR was involved in the functional annotation of the genes.

### Conflict of interest statement

The authors declare that the research was conducted in the absence of any commercial or financial relationships that could be construed as a potential conflict of interest.
