# Supplementary material for: Genomic Diversity and Evolution of the Fish Pathogen Flavobacterium psychrophilum
Source: Front Microbiol. 2018 Feb 7;9:138. doi: 10.3389/fmicb.2018.00138 (PMC5808330; doi:10.3389/fmicb.2018.00138)
Supplement: Supplementary file 1 [file Table1.pdf]

Supplementary Table 1. Sequencing facts for new genomes. [a] 1x and 2x stands for single reads and paired-end reads, respectively. [b] Total number of reads. Reads in both directions are counted in the case of paired-end sequencing. [c] Expected coverage assuming a genome of length 2,860,382 bp (JIP 02/86).

**Isolate**	**Illumina read characteristics**[a]	**Total number of reads (#R1+#R2)**[b]	**expected coverage**[c]
KU 061128-1	1x35 bp	27,572,728	337
KU 060626-59	1x35 bp	28,535,256	349
KU 051128-10	1x35 bp	29,880,537	366
KU 060626-4	1x35 bp	29,914,313	366
LVDJ XP189	1x50 bp	7,587,203	133
JIP 16/00	1x50 bp	7,731,664	135
FPC 831	1x50 bp	8,716,590	152
FPC 840	1x50 bp	8,970,893	157
JIP 08/99	1x50 bp	9,239,171	162
NO042	2x72 bp	20,316,078	511
FI055	2x72 bp	25,247,064	636
FI146	2x72 bp	31,155,716	784
NCIMB1947^{T}	2x72 bp	32,168,372	810
DK001	2x72 bp	32,726,470	824
IT09	2x72 bp	32,959,928	830
CH1895	2x72 bp	33,320,390	839
FI056	2x72 bp	34,283,164	863
NO004	2x72 bp	35,148,424	885
NO098	2x72 bp	36,745,540	925
FI166	2x72 bp	36,865,496	928
LM-02-Fp	2x72 bp	37,351,730	940
DK002	2x72 bp	37,888,744	954
CH8	2x72 bp	38,161,900	961
LM-01-Fp	2x72 bp	38,339,654	965
IT02	2x72 bp	38,359,176	966
DK095	2x72 bp	40,944,934	1,031
DK150	2x72 bp	42,728,890	1,076
FI070	2x72 bp	48,827,910	1,229
NO083	2x72 bp	54,154,230	1,363
FRGDSA 1882/11	2x100 bp	81,061,434	2,834
