# Supplementary material for: Genomic Diversity and Evolution of the Fish Pathogen Flavobacterium psychrophilum
Source: Front Microbiol. 2018 Feb 7;9:138. doi: 10.3389/fmicb.2018.00138 (PMC5808330; doi:10.3389/fmicb.2018.00138)
Supplement: Supplementary file 3 [file Presentation1.pdf]

Supplementary Material

Genomic Diversity and Evolution of the Fish Pathogen *Flavobacterium psychrophilum*

Eric Duchaud, Tatiana Rochat, Christophe Habib, Paul Barbier, Valentin Loux, Cyprien Guérin, Inger Dalsgaard, Lone Madsen, Hanne Nilsen, Krister Sundell, Tom Wiklund, Nicole Strepparava, Thomas Wahli, Greta Caburlotto, Amedeo Manfrin, Gregory Wiens, Erina Fujiwara-Nagata, Ruben Avendaño-Herrera, Jean-François Bernardet, Pierre Nicolas

*** Correspondence:**
Corresponding authors
eric.duchaud@inra.fr, pierre.nicolas@inra.fr

Contents:

Supplementary Table 1. Sequencing facts for new genomes. [Provided as a separate .pdf file]

Supplementary Table 2. Genes specific to CC-ST10. [Provided as a separate .xlsx file]

Supplementary Figure 1. Large inversion around the replication terminus.

Supplementary Figure 2. Trees built on AT-profiles for CC-ST10 isolates.

Supplementary Figure 3. Trees for CC-ST10 obtained by parsimony method on 144 SNPs located outside of recombination tracts.

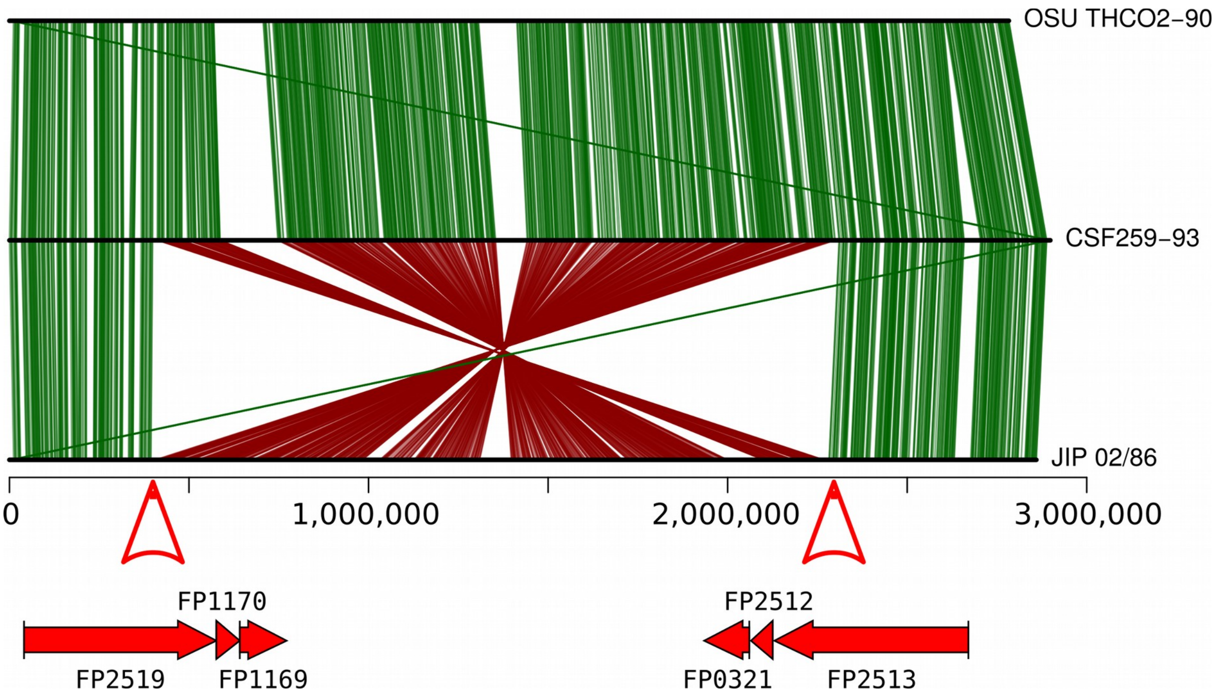

Supplementary Figure 1. Large inversion around the replication terminus. Oblique lines show the respective positions of the 1,549 single copy genes in the three genomes (JIP 02/86, CSF259-93 and OSU THCO2-90) for which assembly has been confirmed by optical mapping for these three genomes. Dark green indicates conserved orientation; Dark red indicates inverted orientation. The inverted region is bordered by three repeated genes of cumulated length ~10 Kbp shown in red; locus tags correspond to those of JIP 02/86.

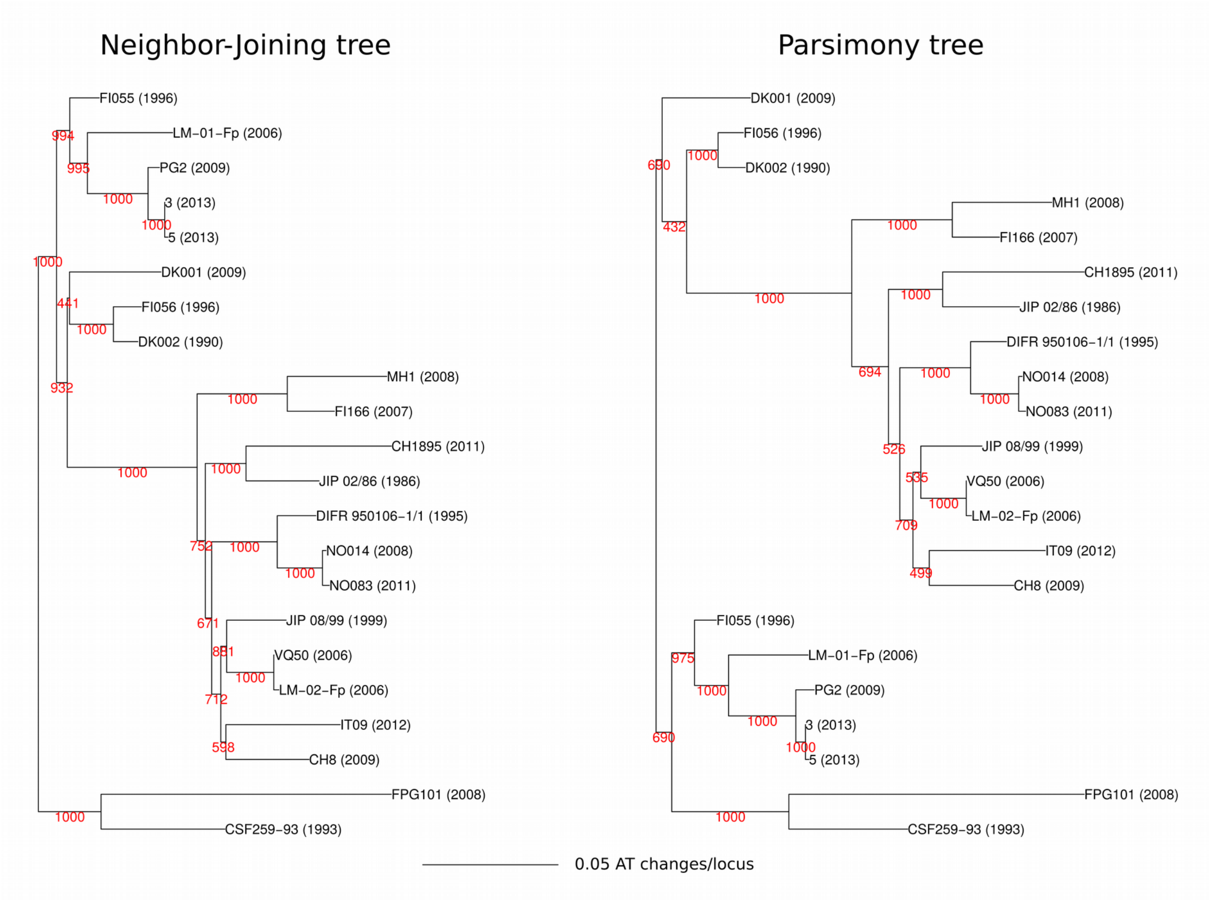

Supplementary Figure 2. Trees built on AT-profiles for CC-ST10 isolates. Two trees are compared: neighbor joining and parsimony. Midpoint rooting is used. The bootstrap support obtained after tree reconstruction on 1000 re-sampled AT-profiles is shown in red.

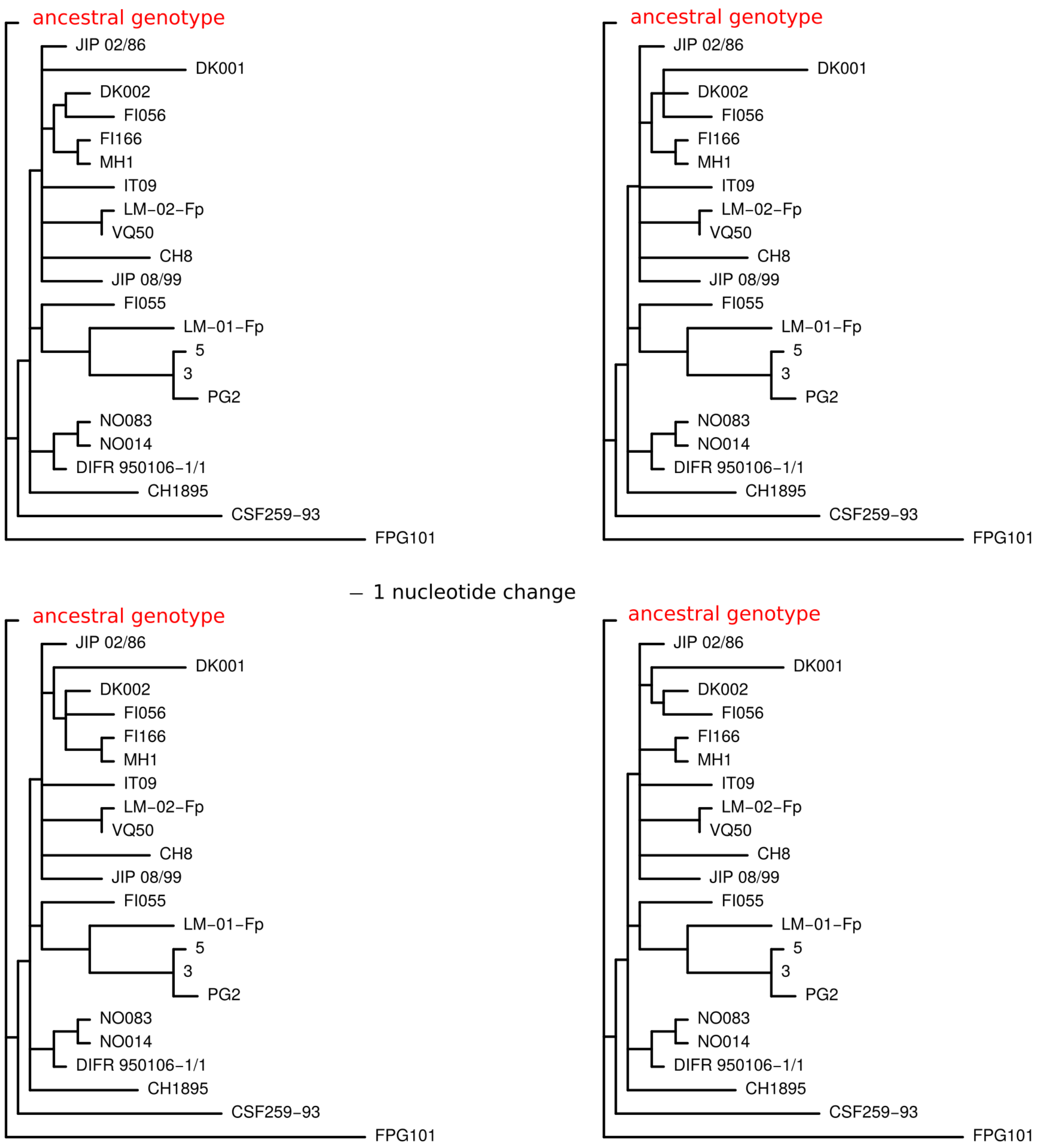

Supplementary Figure 3. Trees for CC-ST10 obtained by parsimony method on 144 SNPs located outside of recombination tracts. A cut-off of 0.25 on the probability to be in a recombination tract across all pairs was applied to select these SNPs. Four equally parsimonious trees were found and are represented, each requiring a total of 147 nucleotide changes. The label “ancestral genotype” in red, corresponding to the genotype with ancestral alleles at the 144 SNPs, was included to give insights onto the position of the root of the CC-ST10 genealogy.
